# Correlation between head tremble and the severity of Parkinson’s disease

**DOI:** 10.1111/cns.13753

**Published:** 2021-11-10

**Authors:** Jingsheng Yang, Quanhao Guo, Xianwei Zou, Meng Wang, Yanxia Wen, Xiaqing Chen, Xiechuan Weng, Fan Xu

**Affiliations:** ^1^ Department of Public Health Chengdu Medical College Sichuan China; ^2^ MOEMIL Laboratory School of Optoelectronic Science and Engineering University of Electronic Science and Technology of China Chengdu China; ^3^ Department of Neurology First Affiliated Hospital of Chengdu Medical College Sichuan China; ^4^ Beijing Institute of Basic Medical Sciences Beijing China

**Keywords:** disease severity, head movement, Parkinson's disease, phonation test

## Abstract

**Introduction:**

Parkinson's (PD) is a common degenerative disease of the central nervous system. It affects more than 6 million individuals worldwide. The typical clinical manifestations include static tremor, slow movement, and unstable posture. However, the correlation between head tremor and the severity of PD remains unclear.

**Methods:**

In the current study, 18 patients and 18 healthy subjects were recruited to undergo a phonation test. Noldus facereader 7.0 software was used to analyze the range of head trembling between the two groups.

**Results:**

The data revealed that patients with PD had significant differences in the x‐, y‐, and z‐axis of head movement with respect to the specific pronunciation syllables compared with the normal group. Moreover, the head movement of the patients with PD was positively correlated with the severity of the disease in the single, double, and multiple syllable tests. In the phonetic test, the head displacement of patients with PD was significantly greater than that of healthy individuals, and the displacement range was positively correlated with the severity of the disease.

**Conclusion:**

These pieces of evidence suggested that the measurement of head displacement assists the early diagnosis and severity of the disease.

## Graphical abstract

The patients with PD had significant differences in the *X*‐, *Y*‐, and *Z*‐axis of head movement with respect to the specific pronunciation syllables compared to the normal group. Moreover, the head movement of the patients with PD was positively correlated with the severity of the disease in the single, double, and multiple syllable tests. In the phonetic test, the head displacement of patients with PD was significantly greater than that of healthy individuals, and the displacement range was positively correlated with the severity of the disease.

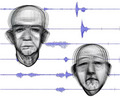

## 1 BACKGROUND

Parkinson's disease (PD) is the second most common neurodegenerative disease and its prevalence has been projected to double over the next 30 years. Linder et al. reported an annual incidence of 18.8 cases/100,000, and the mean age of onset was 70.6 years in Sweden.^1^ Previous studies identified about 150,000 prevalent cases (prevalence = 2.30/1000 population) and 25,000 incident cases (incidence = 0.39/1000 person‐years) in 2010 in France.^2^ Furthermore, 680,000 individuals with PD, aged ≥45‐year‐old, were recorded in 2010, and the number rose to approximately 930,000 in 2020 and will rise to 1,238,000 in 2030 based on the US Census Bureau population projections.^3^ In China, a multiethnic developing country with the largest population in the world is stepping into an aging era.^4^ The average prevalence of PD in China was about 3.8756% in Han population (≥50‐year‐old).^5^ It is estimated that by 2030, Chinese PD patients will increase to 4.94 million, accounting for half of the worldwide PD patients.^6^

The upcoming demographic shift toward older individuals in western nations would require efforts to project the healthcare burden over the coming decades, especially for diseases, such as Alzheimer's disease and PD, with increasing incidence corresponding to age. The projected increase in dementia has been referred to as a “rising tide,” emphasizing the sheer volume of this problem and warnings of public health challenges. Similarly, in China, the population of neurodegenerative disease will increase over time and cast a huge burden on the economics and healthcare system.

The body posture of the patient becomes stooped, and axial and limb rigidity with or without cogwheel phenomenon is noted with a tendency for a shuffling gait and lack of arm swing while walking. Bradykinesia may lead to expressionless face (hypomimia), and the amplitudes of handwriting become small (micrographia). About 80% of the limbs have tremors, and most commonly, a resting pill‐rolling type of tremor in the hands. The pill‐rolling relates to the tendency of the contact between the thumb and the index finger and perform a circular movement (Jankovic).^7^

Dystonia is a motor symptom in PD. It describes a sustained muscular contraction frequently accompanied by abnormal movements, postures, or both. This might rarely be a prediagnostic symptom in PD. The typical prediagnostic dystonias include unilateral equinovarus foot position, upper arm‐forearm or forearm‐hand flexion, writer's cramp, oromandibular dystonia, torticollis, or different combinations of these symptoms (Tolosa and Compta).^8^ Rigidity in PD means stiff, inflexible, and painful muscles all over the body but prominently in the trunk, head, and extremities, along with regular cramps and pain. Thus, rigidity is one of the primary features of PD.^9^

With the progression of PD, patients experience varied speech problems, such as hoarseness and weakness, during communication. Also, the normal speech of PD patients is altered. The most commonly reported speech problems are sudden fast or slow speech, hoarseness, imperfect talk, monotonous or nasal voice, tremorous and stuttering speech, and also difficulty in initiating the speech.^10^

In the phonetic test, the head displacement of PD patients was significantly greater than that of healthy individuals, and the displacement amplitude was positively correlated with the severity of the disease. Furthermore, the measurement of head displacement assisted the early diagnosis and severity of the disease. Presently, only a few studies have reported the correlation between the severity of PD and the early cranial tremor. Break et al. hypothesized that the pathological staging of PD can be divided into six stages. In stages 3 and 4, substantia nigra, deep mesencephalic nuclei, and anterior cerebral lobes were involved, resulting in the motor symptoms of PD. The specificity of early olfactory disorders, sleep disorders, and other non‐motor symptoms could lead to missed clinical diagnosis. Conversely, head shaking is a specific motor symptom. This study aimed to decipher an accurate degree of head shaking to help the early recognition of PD as an auxiliary diagnosis in the clinic.

## 2 METHODS

### 2.1 Ethics statement

The study was approved by the Institute of Institutional Review Board and by the Ethics Committee of Chengdu Medical College. All participants provided written informed consent.

### 2.2 Participants

A total of 18 PD patients admitted to the Neurology Department of the First Affiliated Hospital of Chengdu Medical College were selected from January 2020 to February 2021, and 18 healthy subjects comprised the control group. The course of the disease was recorded, and the severity of PD was assessed using Hoehn‐Yahr (HY) scale and Unified Parkinson's Disease Rating Scale (UPDRS III). Patients with PD fulfilled the following inclusive criteria^1^: idiopathic PD and no other neurological defects^2^; movement fluctuations that respond satisfactorily to L‐dopa (“ON‐OFF” effect) ^3^; no previous psychiatric problems or cognitive decline.

### 2.3 Measurement of head tremor during the phonation test

The candidates were invited to participate in the phonation test in a light‐stable, suitable, and quiet room. The vowels “a,” “o,” and “e” were selected to form the monosyllable, double syllable, and multiple syllables. When the test sample was pronounced, the airflow from the lungs strike the vocal cords through the glottis, making them vibrate evenly. For more details of the phonation test cards, see the online supporting file. Then, the trill air flows through the mouth and makes different sounds through the adjustment of the tongue and lips. A detailed phonation test protocol was utilized as described previously^11^ as an active exercise that coordinates the movements of other parts. Consequently, the built‐in camera in the laptop was used to record the head tremor and detailed facial expressions. Finally, Facereader 7.0 software was used to analyze the head tremor.^12^

### 2.4 Statistical analysis

The data were recorded and stored in Excel files, and Stata 15.0 was used for statistical analysis. The HY scale and UPDRS III were expressed as the mean (standard deviation) and analyzed by two‐tailed Student's *t*‐test. Also, age was compared between the two groups using two‐tailed Student's *t*‐test. Sex, profession, alcohol consumption, and education were compared using the chi‐square test. The head tremor between the PD patients and healthy subjects was compared using two‐tailed Student's *t*‐test. Spearman's rank correlation coefficient was used to analyze the correlation between head tremor and PD severity. *p* < 0.05 indicated statistically significant difference.

## 3 RESULTS

### 3.1 Demographic characteristics

A total of 18 PD patients and 18 HC subjects were recruited in this study. Age was similar between the two groups (*t* = 0.9005, df = 34, *p* = 0.8129), while sex distribution was balanced in the two groups (χ^2^ = 3.01, *df* = 1, *p* = 0.083). In the PD group, the duration of disease was 4.98 ± 4.38 years, and the average H&Y stage and UPDRS III score were 2.42 ± 0.56 and 36.00 ± 15.28, respectively. No significant difference was observed in alcohol consumption (χ^2^ = 2.2154, *df* = 1, *p* = 0.14), smoking habits (χ^2^ = 0.8000, *df* = 1, *p* = 0.37), profession (χ^2^ = 1.1246, *df* = 2, *p* = 0.57), and education level (χ^2^ = 0.2476, *df* = 3, *p* = 0.97) between the two groups (Table 1).

**TABLE 1:** Baseline characteristics of the participants

Variables	PD	Control	*p*‐value
*N*	18	18	
Age*	72.67 (7.80)	70.11(9.20)	0.37
Sex (M/F) #	9/9	4/14	0.08
Duration of disease	4.98 (4.38)	–	
HY	2.42 (0.65)	–	
UPDRS III	36.00 (15.28)	–	
Alcohol consumption # (N/Y)	11/7	15/3	0.14
Smoker # (N/Y)	14/4	16/2	0.37
**Profession#**			0.57
Retired	6	4	
Farmer	10	13	
Worker	2	1	
**Education #**			0.97
Primary school	10	11	
Middle school	3	3	
High school	3	2	
Master's	2	2	

^#^Chi‐square test.

*Two‐tailed Student's *t*‐test.

### 3.2 Head tremor in male patients with PD and control groups

Among the 26 phonation test samples, significant differences were detected in 1–, 9–17, and 19–26 syllables between the PD and control groups in the x‐axis trajectory direction (*p* < 0.05). However, no significant differences were found in 8 and 18 syllables (*p* > 0.05). In addition, we found significant differences in 1, 4, 6, 7, 9, 10, 12, 13, 15, 18, 20–22, and 24–26 syllables between PD patients and normal people in the y‐axis trajectory direction (*p* < 0.05). On the other hand, no significant difference was observed in 2, 3, 5, 8, 11, 14, 16, 17, 19, and 23 syllables (*p* > 0.05). Moreover, the data displayed significant differences in the 2–13, 15–21, and 24–26 syllables between PD patients and normal individuals in the z‐axis trajectory direction (*p* < 0.05), while no significant difference was detected in 1, 14, 22, and 23 syllables between the two groups (*p* > 0.05) (Table 2).

**TABLE 2:** Head tremor in male patients with PD and control groups in phonetic test

Speech samples	x‐axis motion track				y‐axis motion track				z‐axis motion track			
	PD		Control		PD		Control		PD		Control	
	Mean	SD	Mean	SD	Mean	SD	Mean	SD	Mean	SD	Mean	SD
1	5.09*	8.55*	−4.96*	10.75*	0.40*	5.07*	−1.97*	10.47*	−0.95	4.24	−0.62	5.48
2	3.85*	6.01*	0.81*	5.69*	−0.55	3.56	−0.56	15.73	−1.11*	4.66*	−4.22*	6.90*
3	3.86*	5.86*	0.80*	4.08*	0.39	4.35	−0.70	14.54	−0.25*	3.86*	−4.48*	6.55*
4	3.16*	7.13*	1.65*	4.21*	2.84*	5.53*	1.50*	13.09*	−0.64*	4.44*	−4.75*	7.29*
5	5.58*	7.95*	2.53*	9.73*	1.87	4.65	3.37	14.00	−0.21*	3.24*	−2.78*	5.50*
6	3.6*	7.40*	0.51*	7.21*	2.63*	6.95*	−1.43*	14.60*	−0.72*	3.98*	−1.70*	7.64*
7	4.77*	6.33*	2.34*	8.80*	2.01*	10.48*	−0.04*	13.44*	−1.69*	4.34*	−2.38*	6.73*
8	3.98	5.16	3.80	7.21	2.63	8.67	5.24	14.58	−3.06*	5.19*	0.97*	8.97*
9	4.01*	6.06*	0.59*	5.20*	1.26*	5.66*	−0.43*	16.23*	−2.54*	5.05*	−4.41*	6.73*
10	5.16*	5.45*	0.11*	4.75*	1.63*	6.08*	−0.62*	15.33*	−1.65*	4.63*	−4.69*	6.85*
11	4.68*	5.17*	−0.79*	3.56*	0.04	6.44	1.71	12.60	−0.83*	4.27*	−3.82*	6.99*
12	4.86*	4.95*	1.87*	5.73*	0.61*	6.11*	4.39*	13.24*	−1.00*	4.54*	−6.54*	8.21*
13	4.47*	4.84*	−0.16*	9.34*	0.14*	6.83*	4.4*	10.8*	−0.95*	3.91*	1.25*	8.75*
14	4.04*	4.89*	−1.23*	7.23*	1.99	6.22	0.78	12.63	−0.62	3.46	−1.08	8.20
15	4.87*	4.79*	−2.32*	6.15*	0.94*	5.82*	2.89*	12.67*	−0.94	3.86	−1.75*	8.75*
16	4.39*	4.58*	−4.34*	9.16*	0.33	8.38	1.49	13.81	−1.07	3.82	−1.91*	9.55*
17	3.48*	4.72*	0.62*	7.58*	1.91	8.11	2.17	14.56	−0.51	3.38	−3.69*	11.14*
18	3.46	3.86	3.56	11.29	0.58*	6.81*	5.56*	9.82*	−0.36	3.44	2.24*	8.06*
19	2.89*	4.02*	−2.44*	6.73*	0.28	7.59	0.77	12.76	−0.10	3.54	−2.52*	9.53*
20	6.23*	5.28*	−0.02*	6.25*	2.58*	5.41*	1.27*	12.91*	0.26	2.39	−3.25*	11.33*
21	4.64*	5.48*	−2.19*	6.46*	1.01*	6.03*	3.41*	11.95*	0.11	3.13	−1.63*	11.42*
22	3.53*	8.72*	−3.01*	5.66*	1.32*	5.68*	−2.00*	13.94*	0.13	3.43	−0.33	7.13
23	3.66*	4.88*	−3.70*	8.13*	2.31	8.28	1.70	11.62	−0.37	3.62	−0.73	7.77
24	3.86*	4.77*	−3.66*	6.46*	1.55*	6.86*	−2.73*	14.28*	−1.03	4.25	−2.12*	8.77*
25	3.34*	4.86*	−0.40*	6.37*	−0.14*	5.60*	3.49*	10.94*	0.07	3.43	−3.37*	11.39*
26	4.64*	9.90*	−4.84*	10.62*	1.72*	6.92*	−0.71*	11.31*	0.18	3.84	−0.66*	7.63*

Note

Two‐tailed Student's *t* test.

*p < 0.05.

### 3.3 Head tremor in female patients in the PD and control groups

Among the 26 phonation test samples, significant differences were observed in the 2–24, 26 syllables between the PD group and the controls in the x‐axis trajectory direction (*p* < 0.05), while no significant difference was detected in 1 and 25 syllables (*p* > 0.05). Furthermore, significant differences were detected in all the 26 syllables between the PD group and the controls in the y‐axis trajectory direction (*p* < 0.05). Moreover, significant differences were observed in the 3–9, 12–14, 16, 19, 21–24, and 26 syllables between the PD group and controls in the z‐axis trajectory direction (*p* < 0.05). Similarly, no significant difference was found in 1, 2, 10, 11, 15, 17, 18, 20, and 25 syllables between the two groups (*p* > 0.05) (Table 3).

**TABLE 3:** Head tremor in female patients with PD and control group in phonetic test

Speech samples	x‐axis motion track				y‐axis motion track				z‐axis motion track			
	PD		Control		PD		Control		PD		Control	
	Mean	SD	Mean	SD	Mean	SD	Mean	SD	Mean	SD	Mean	SD
1	6.72	10.14	7.12	9.48	1.17*	10.58*	−5.59*	8.61*	−2.83	3.13	−2.82	5.88
2	7.82*	14.46*	1.60*	8.10*	4.04*	13.51*	−7.17*	9.44*	−2.98	3.98	−2.46	6.50
3	3.16*	7.72*	−1.26*	5.64*	2.42*	13.82*	−8.34*	9.38*	−2.65*	3.37*	0.00*	6.19*
4	4.8*	9.67*	−0.66*	10.06*	1.52*	12.80*	−4.89*	8.11*	−2.46*	3.40*	−1.48*	6.74*
5	7.18*	10.69*	−1.18*	5.99*	0.60*	10.70*	−6.85*	7.93*	−2.64*	3.22*	−0.40*	6.95*
6	7.98*	9.47*	−1.33*	5.91*	0.18*	6.98*	−5.22*	9.03*	−3.27*	2.89*	−0.82*	6.38*
7	7.08*	10.41*	−1.46*	6.30*	0.79*	6.66*	−6.52*	8.29*	−3.25*	3.16*	−1.02*	7.13*
8	6.88*	8.03*	1.65*	9.25*	1.62*	5.49*	−4.89*	12.23*	−3.17*	3.05*	−0.80*	6.11*
9	5.76*	7.03*	0.38*	6.79*	2.3*	7.50*	−4.51*	7.41*	−2.57*	3.78*	−0.63*	6.61*
10	5.65*	6.40*	−0.19*	6.88*	4.14*	9.34*	−6.19*	7.86*	−1.94	3.91	−1.38	6.91
11	5.71*	7.51*	−0.16*	6.96*	3.40*	8.33*	−5.3*	8.17*	−1.86	4.29	−1.46	7.88
12	4.74*	4.16*	−0.96*	6.71*	0.65*	5.89*	−6.24*	8.33*	−2.53*	3.40*	−0.75*	7.77*
13	7.14*	8.52*	0.47*	6.93*	−0.67*	7.01*	−6.01*	7.70*	−2.86*	3.83*	−1.25*	7.30*
14	6.25*	9.11*	1.47*	9.07*	−0.64*	8.31*	−7.70*	8.95*	−2.74*	4.12*	−1.36*	7.47*
15	5.45*	9.08*	0.56*	6.99*	−2.23*	7.75*	−6.26*	7.46*	−1.69	4.31	−1.77	7.18
16	4.67*	6.52*	0.43*	6.83*	−0.39*	5.96*	−5.62*	7.61*	−2.58*	4.00*	−1.26*	7.98*
17	6.55*	8.09*	0.35*	7.76*	1.11*	5.65*	−8.57*	9.33*	−2.90	4.25	−2.57	7.96
18	5.98*	8.35*	0.41*	8.96*	1.17*	8.59*	−8.66*	8.35*	−2.01	4.85	−1.78	7.01
19	4.97*	7.47*	1.59*	8.49*	−0.40*	7.99*	−8.51*	7.48*	−2.80*	4.50*	−1.25*	7.01*
20	4.37*	5.72*	2.89*	10.28*	0.88*	11.44*	−6.61*	7.64*	−1.45	5.39	−1.63	6.68
21	3.80*	4.80*	−0.24*	7.77*	0.48*	9.15*	−6.88*	9.58*	−1.81*	4.63*	−0.75*	8.19*
22	4.51*	7.39*	−0.63*	7.18*	0.12*	8.43*	−9.67*	10.14*	−2.28*	3.96*	−0.14*	7.96*
23	5.66*	9.06*	−0.51*	6.61*	1.88*	6.91*	−7.41*	9.22*	−2.21*	4.63*	0.68*	6.17*
24	4.49*	5.62*	0.95*	7.26*	2.24*	8.29*	−8.12*	7.80*	−1.63*	4.76*	−1.12*	5.94*
25	3.25	4.64	2.62	11.12	4.74*	9.51*	−7.34*	9.00*	−1.93	3.97	−2.08	8.60
26	0.24*	6.12*	0.98*	9.47*	4.70*	9.81*	−4.38*	8.00*	−2.22*	3.48*	−0.98*	7.02*

Note

Two‐tailed Student's *t* test.

*p < 0.05.

### 3.4 Correlation between head tremor and disease severity in PD males in phonetic tests

The data established a positive or negative correlation between head tremor in x‐/y‐/z‐axis and the severity of PD during the different phonation tests among male subjects. Regarding the HY scale, the coefficient between head tremor in z‐axis, and HY severity scale was 0.1919 in a single syllable (*p* < 0.05). Interestingly, our subjects presented a positive correlation between HY scale and head tremor in x‐/y‐/z‐axis in double syllable test (*p* < 0.05). x‐/y‐axis head tremor showed a positive and negative correlation with HY scale (*p* < 0.05). Also, a positive and negative correlation was established between head tremor in x‐/y‐/z‐axis and UPDRS‐III scale (*p* < 0.05).

### 3.5 Correlation between head tremor and disease severity of female PD in phonetic tests

Similar to males, the female subjects showed a positive or negative correlation between head tremor in x‐/y‐/z‐axis and the severity of PD during various phonation tests. According to the HY scale, the coefficient between head tremor in x‐ and y‐axis and HY severity scale was −0.0878 and 0.0618, respectively, in a single syllable (*p* < 0.05). The subjects in this study presented a negative and positive correlation between HY scale and head tremor in y‐ and z‐axis in double syllables (*p* < 0.05), while only y‐ and z‐axis head tremor presented a negative correlation with HY scale (*p* < 0.05). Similarly, a positive and negative correlation was established between head tremor in x‐/y‐/z‐axis and UPDRS‐III scale, except x‐axis in the double syllable test. (*p* < 0.05).

Tremor is one of the typical motor symptoms of PD. The primary clinical feature of tremor is a postural or action tremor at 5–12 Hz frequency with symmetrical presentation that involves the hands, head (“yes–yes” or “no–no”), and voice.^13^ In this study, the head tremor was stimulated through the phonation test, and the head movement track in the x‐, y‐, and z‐ axis directions were recorded. Tables 2 and 3 showed that Parkinson's patients have a greater amplitude of tremor in the x‐axis direction, and the main form of head movement was “no‐no.” Among the 26 phonation samples in 1, 5, 10, and 20 syllable tests, male Parkinson's patients had greater head tremor. In 2–4, 6, 19, and 22–25 syllable tests, male Parkinson's patients had smaller head tremor (Table 2), while in 2, 5–7, and 13 syllable tests, female Parkinson's patients had greater head tremor. In the 3, 4, 9–12, 15–16, 18–24, and 26 syllable tests, female Parkinson's patients had small head tremor (Table 3). On the x‐axis, the head movement of Parkinson's patients was negatively correlated with the severity of the disease. On the Y and Z axes, the head movement of Parkinson's patients was positively correlated with the severity of the disease, which was related to muscle stiffness.

## 4 DISCUSSION

During the phonation test, patients with PD showed significant differences in the head tremor compared with healthy controls, especially the x‐axis. Several studies demonstrated that tremor is mechanically transmitted from one limb to the other body parts, including the head.^14^, ^15^

According to the study by Roze et al., the head tremor was noticed in 2/5 PD patients as the initial manifestation of PD.^16^ Currently, only a few studies have reported the correlation between the early cranial tremor and the severity of PD worldwide. Herein, we observed significant differences in the head movement of male PD patients and the control group in the 26 syllable test between PD patients and normal people in 1–‐7, 9–17, and 19–26 syllables in the direction of x‐axis movement track (*p* < 0.05), while in the direction of y‐axis movement track, significant differences between PD patients and normal people were noted in 1, 4, 6, 7, 9, 10, 12, 13, 15, 18, 20–22, and 24–26 syllables (*p* < 0.05). Moreover, in the direction of z‐axis movement track, significant differences were detected between PD patients and normal individuals in 2–13, 15–21, and 24–26 syllables (*p* < 0.05; Table 2). The study by Kaski et al. demonstrated that the “ocular tremor” observed in the 2 patients is a compensatory eye movement secondary to transmitted head tremor.^17^ Furthermore, significant differences were noted in 2–24 and 26 syllables between PD patients and normal people in the direction of x‐axis head movement track, as assessed in the 26 syllable test (*p* < 0.05). Conversely, in the direction of y‐axis movement track, significant differences were noted in 1–26 syllables between PD patients and normal individuals (*p* < 0.05). In the direction of z‐axis movement track, significant differences were detected in 3–9, 12–14, 16, 19, 21–24, and 26 syllables between PD and normal people (*p* < 0.05; Table 3).

Duval and Hutchison described the mechanism of central tremor based on basal nucleus thalamus cerebellum cortex using the “induction switch regulation” model. The study suggested that PD tremor was enhanced by the inhibitory output of the inner nucleus of globus pallidus, and the lateral premotor cortex and motor cortex of the ventral extraventral anterior nucleus (5–10 ms) of the thalamus (25–30 ms) were activated. The cerebellum stabilizes the frequency and amplitude of tremor, antagonizing the contraction of the muscles, resulting in the second activation of the somatomotor cortex. Subsequently, the tremor signal is transmitted to the cortex and then to the periphery through the corticospinal tract, causing a limb tremor.^18^

Interestingly, the current data disclosed that the head movement of x‐axis motion track of PD patients is negatively correlated with the severity of the disease in the single, double, and multiple syllable tests. The head movement of y‐axis and z‐axis motion track of PD patients is positively correlated with the severity of the disease in the single, double, and multiple syllable tests. These findings indicated that the motion amplitude of PD patients increases with the aggravation of the disease in the direction of y‐ and z‐axis, and in contrast, the motion amplitude decreases in the direction of x‐axis (Tables 4 and 5).

**TABLE 4:** Correlation between head tremor and disease severity of male PD patients

Syllable		HY		UPDRS Ⅲ	
		Rho	*p*	Rho	*p*
Single	X	−0.0229	0.0593	−0.2065	0.0000*
	Y	−0.0167	0.1701	0.1576	0.0000*
	Z	0.1919	0.0000*	0.3956	0.0000*
Double	X	0.0703	0.0000*	−0.2040	0.0000*
	Y	0.1600	0.0000*	0.3709	0.0000*
	Z	0.1149	0.0000*	0.3695	0.0000*
Multiple	X	−0.1024	0.0000*	−0.2284	0.0000*
	Y	0.0367	0.0189*	0.1896	0.0000*
	Z	−0.0006	0.9669	0.2173	0.0000*

Note

Spearman analysis.

*p < 0.05.

**TABLE 5:** Correlation between head tremor and disease severity of female PD patients

Syllable		HY		UPDRS Ⅲ	
		Rho	*p*	Rho	*p*
Single	X	−0.0878	0.0000*	−0.0902	0.0000*
	Y	0.0618	0.0000*	0.3264	0.0000*
	Z	−0.0141	0.2967	0.1276	0.0000*
Double	X	−0.0048	0.7766	0.0036	0.8299
	Y	−0.1503	0.0000*	0.0334	0.0462*
	Z	0.1623	0.0000*	0.2660	0.0000*
Multiple	X	0.0051	0.7457	0.0470	0.0026*
	Y	−0.0334	0.0325*	0.1938	0.0000*
	Z	−0.0328	0.0358*	0.0680	0.0000*

Note

Spearman analysis.

*p<0.05.

The main neck muscles involved in the head movement include the head splint muscle and sternocleidomastoid muscle. In the x‐axis direction, the right cephalic muscle participates in the head rotation from left to right on the same side. The right sternocleidomastoid muscle participates in the head rotation from right to left on the opposite side.^19^ In the direction of y‐ and z‐axis, the head splint muscle participates in the movement of the head from flexion to the center,^20^ and the sternocleidomastoid muscle participates in the movement of the head from the center to tilt back.^21^

The early clinical symptoms of PD are atypical, and a diagnosis based on the history and clinical manifestations is difficult. However, when the clinical symptoms are obvious, the number of dopamine neurons in substantia nigra decrease by 60%–70%, and the opportunity of early treatment is lost.^22^, ^23^ Previous studies have shown that the treatment of most neurons in the premotor period without degeneration protects the nerves and may delay the clinical progress of PD. Therefore, early diagnosis and early treatment of PD patients is essential.^24^ Break et al.^25^ proposed six stages of PD. In stages 3 and 4, substantia nigra, deep midbrain nuclei, and anterior cerebral lobes are involved, resulting in the motor symptoms of PD. The early non‐motor symptoms of PD, such as olfactory disorders and sleep disorders, are not specific and could be missed easily in the clinic. Conversely, the head tremor belongs specifically to the motor symptoms. Taken together, the present study aimed to provide an accurate degree of head tremor and contribute to the early recognition of PD as an auxiliary diagnosis in the clinic.

## 5 CONCLUSION

In the phonetic test, PD patients showed typical displacement compared with normal individuals, and the displacement amplitude was positively correlated with the severity of the disease. This study determined an accurate degree of cranial tremor, which is valuable for the early detection of PD.

### 5.1 Limitation

The present study has some limitations. First, the sample size was small. Second, although patients in this study stopped levodopa before speech testing, they were still using other anti PD drugs and were in the “on” phase.

## CONFLICT OF INTERESTS

The authors declare that they have no competing interests.

## AUTHOR CONTRIBUTIONS

YJS, WXC, and ZXW developed the study concept and drafted the manuscript with XF; WM, WYX, and CXQ recruited the subjects and collected the phonation test and facial expression data; WXC, YJS, and WM performed the Noldus facereader 7.0 operation; CXQ and WXC conducted the statistical analysis with XF.

## Supporting information

Supplementary Material

## Data Availability

All data would be made available upon reasonable request.
