# Supplementary material for: Correlation between head tremble and the severity of Parkinson’s disease
Source: CNS Neurosci Ther. 2021 Nov 10;28(2):218–25. doi: 10.1111/cns.13753 (PMC8739048; doi:10.1111/cns.13753)
Supplement: Supplementary file 1 — Supplementary Material [file CNS-28-218-s001.pptx]

## Slide 1
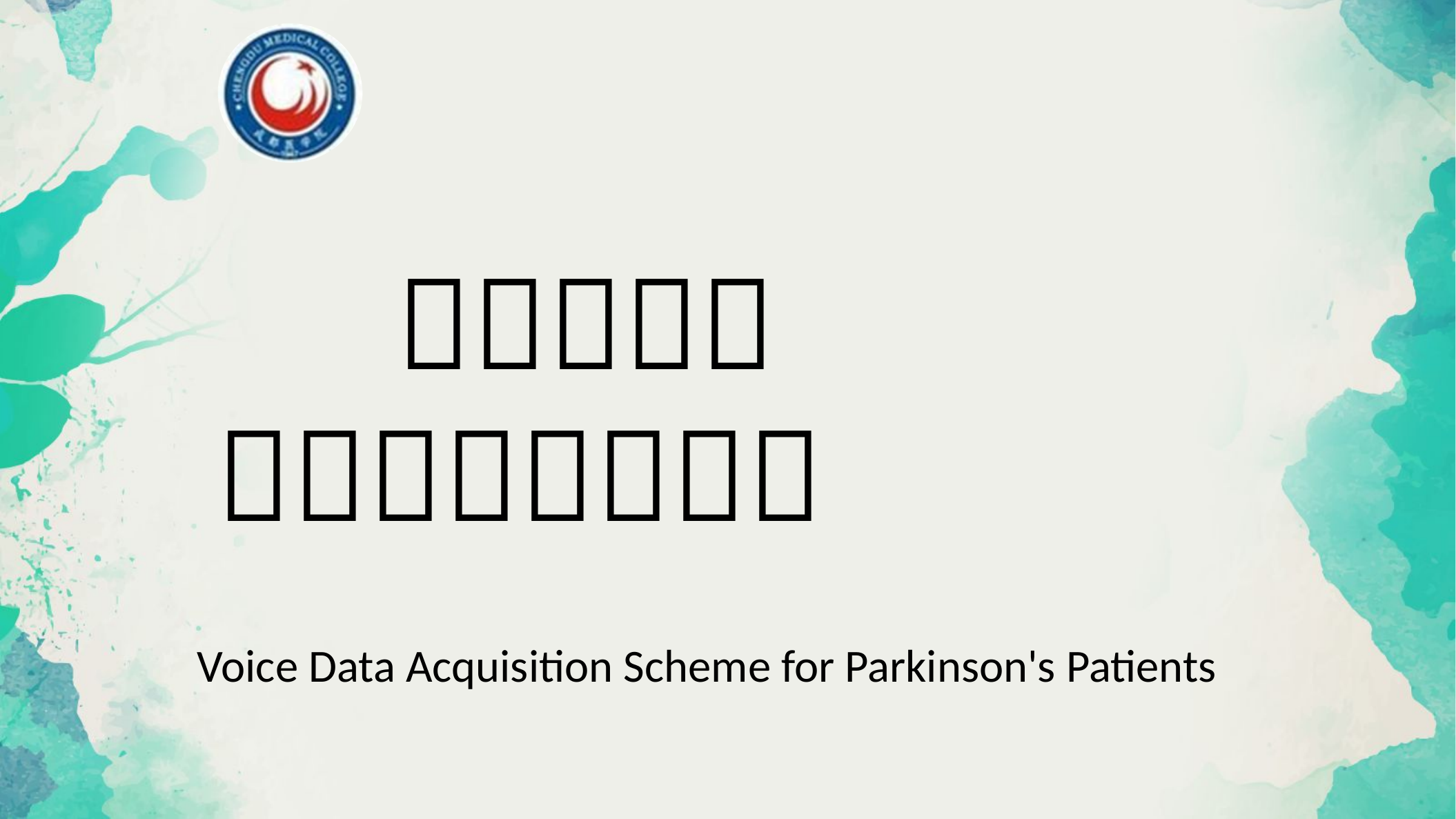

帕金森病人
声音数据采集方案
Voice Data Acquisition Scheme for Parkinson's Patients

## Slide 2
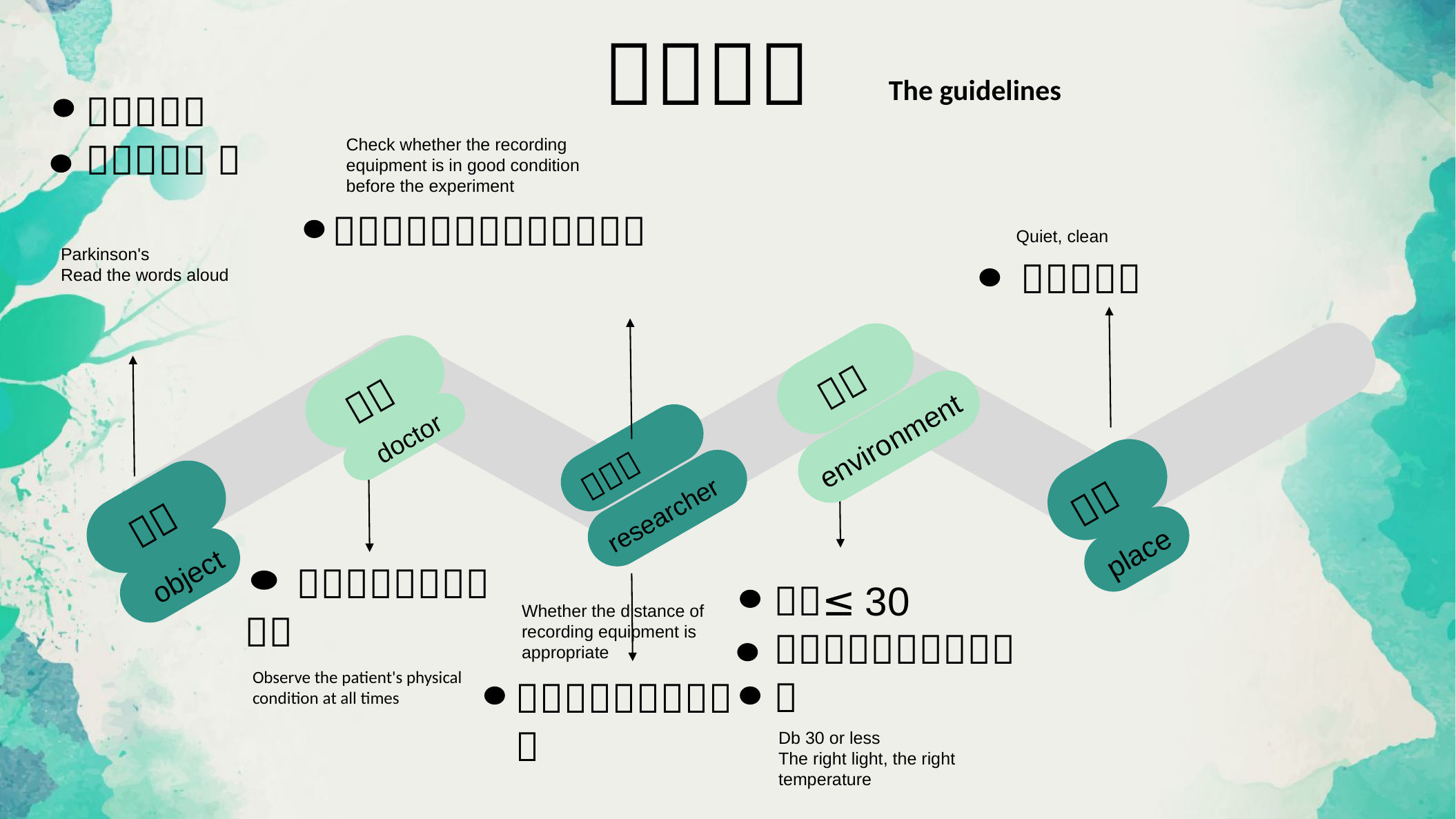

实验须知
The guidelines
 帕金森病人
 大声朗读字 词
Check whether the recording equipment is in good condition before the experiment
 环境
 医生
研究员
场所
 对象
实验前检查录音设备是否完好
 Quiet, clean
Parkinson's
Read the words aloud
 安静，干净
environment
 doctor
researcher
place
 object
 随时观察病人身体状况
分贝≤30
适宜的光线，合适的温度
Whether the distance of recording equipment is appropriate
Observe the patient's physical condition at all times
录音设备距离是否合适
Db 30 or less
The right light, the right temperature

## Slide 3
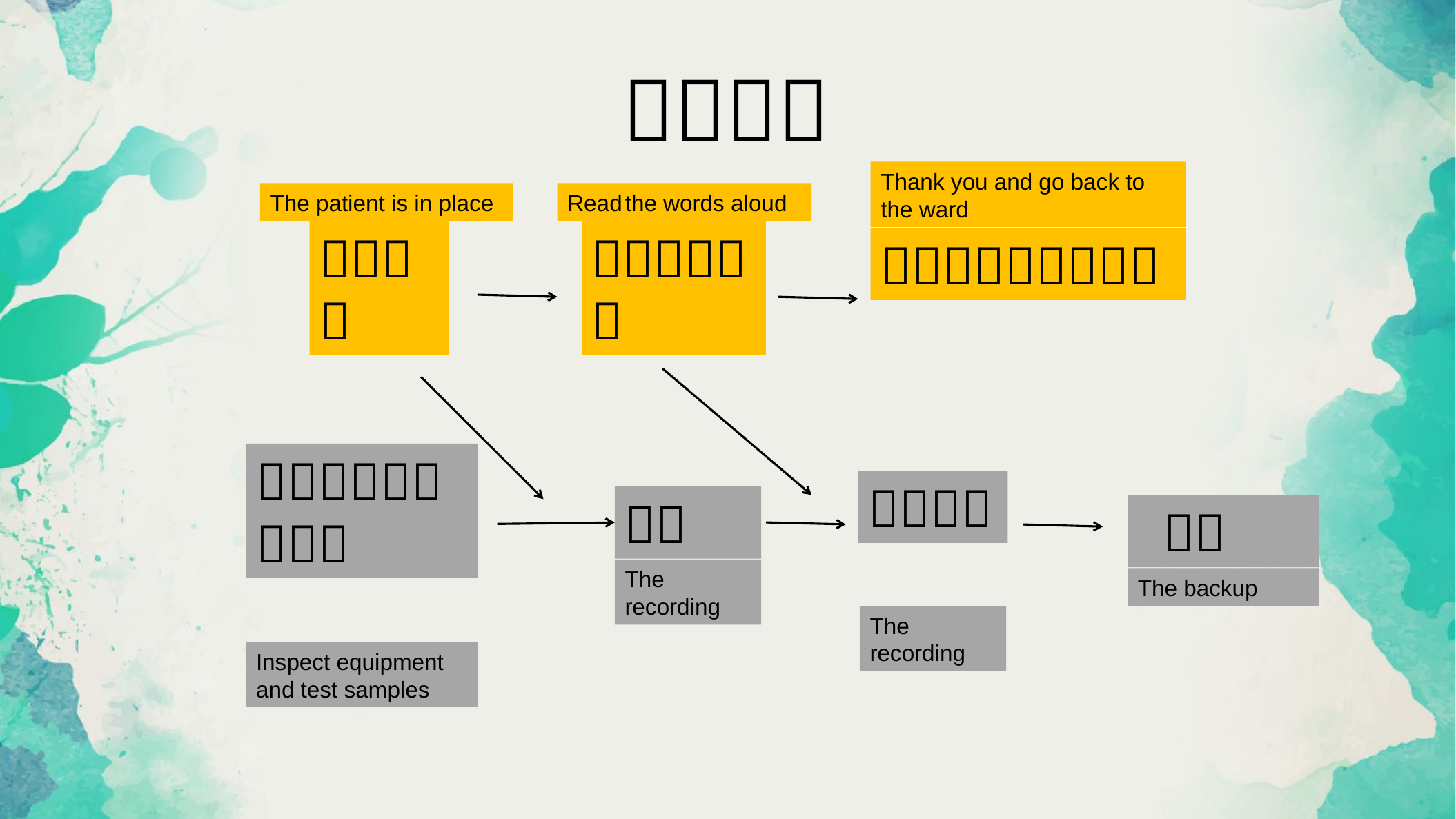

# 实验流程
Thank you and go back to the ward
The patient is in place
Read the words aloud
大声朗读字词
病人就位
表达感谢，回到病房
检查设备和测试样本
录入数据
录音
 备份
The recording
The backup
The recording
Inspect equipment and test samples

## Slide 4
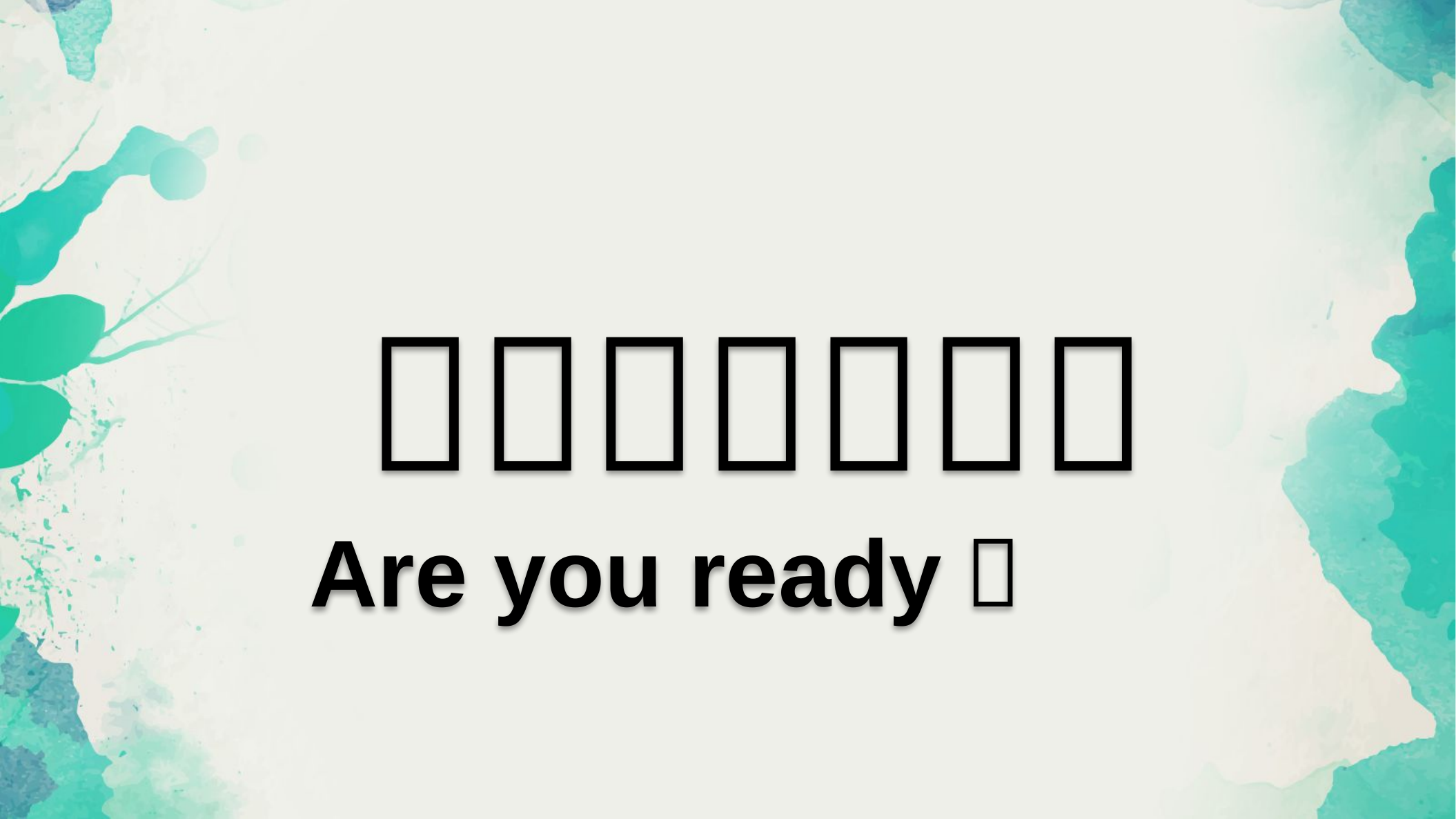

你准备好了吗？
Are you ready？

## Slide 5
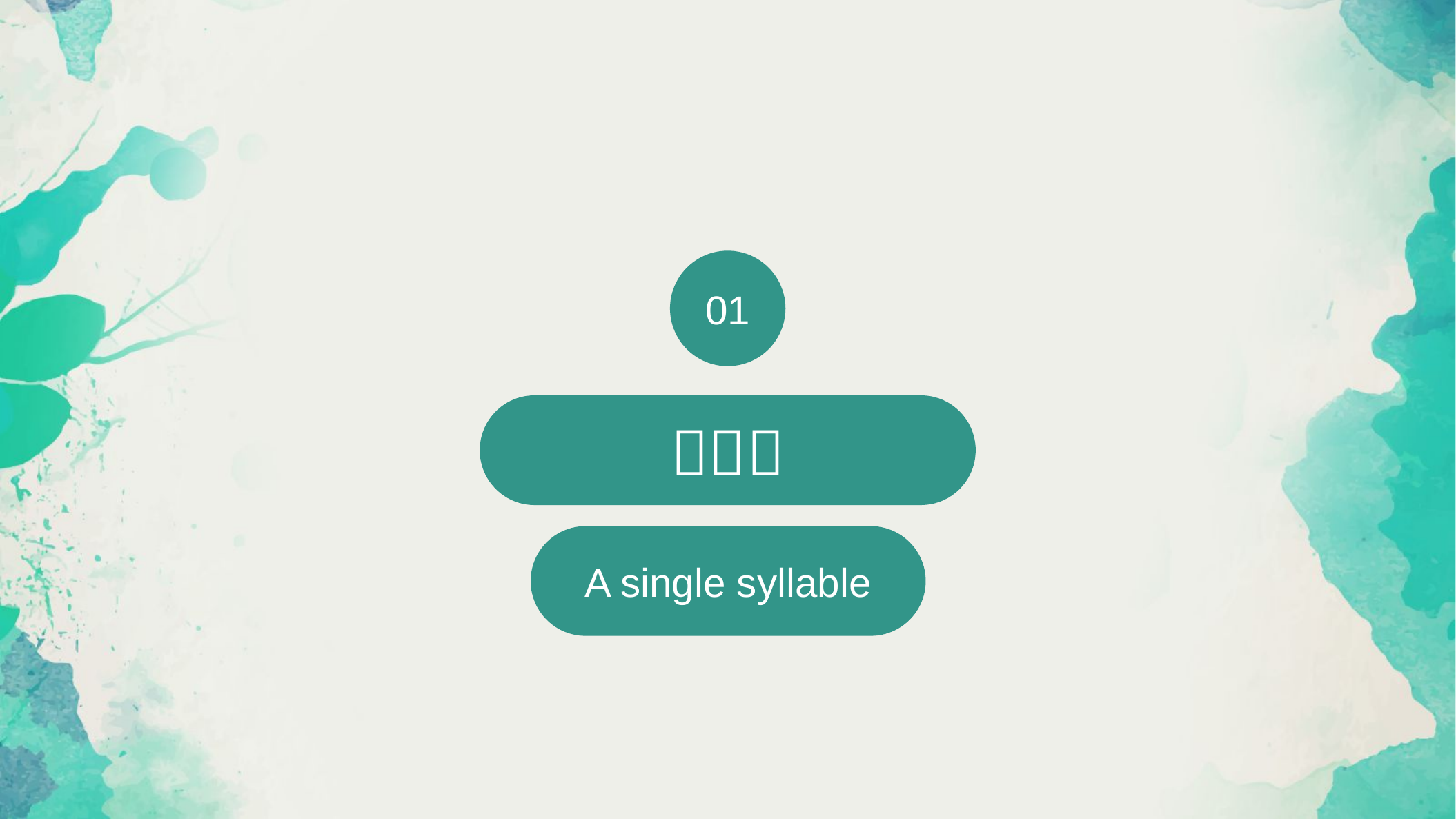

01
单音节
A single syllable

## Slide 6
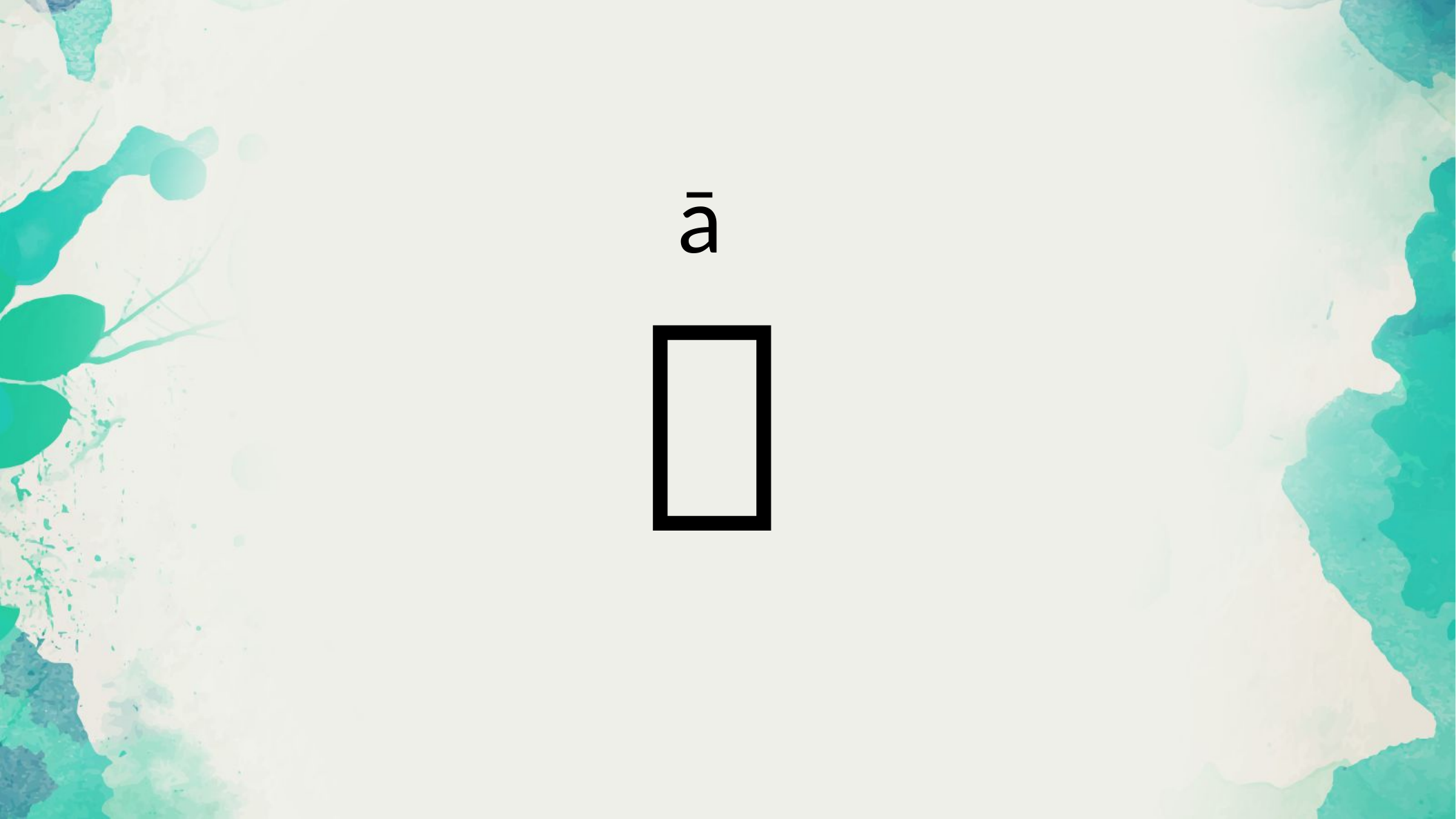

ā
阿

## Slide 7
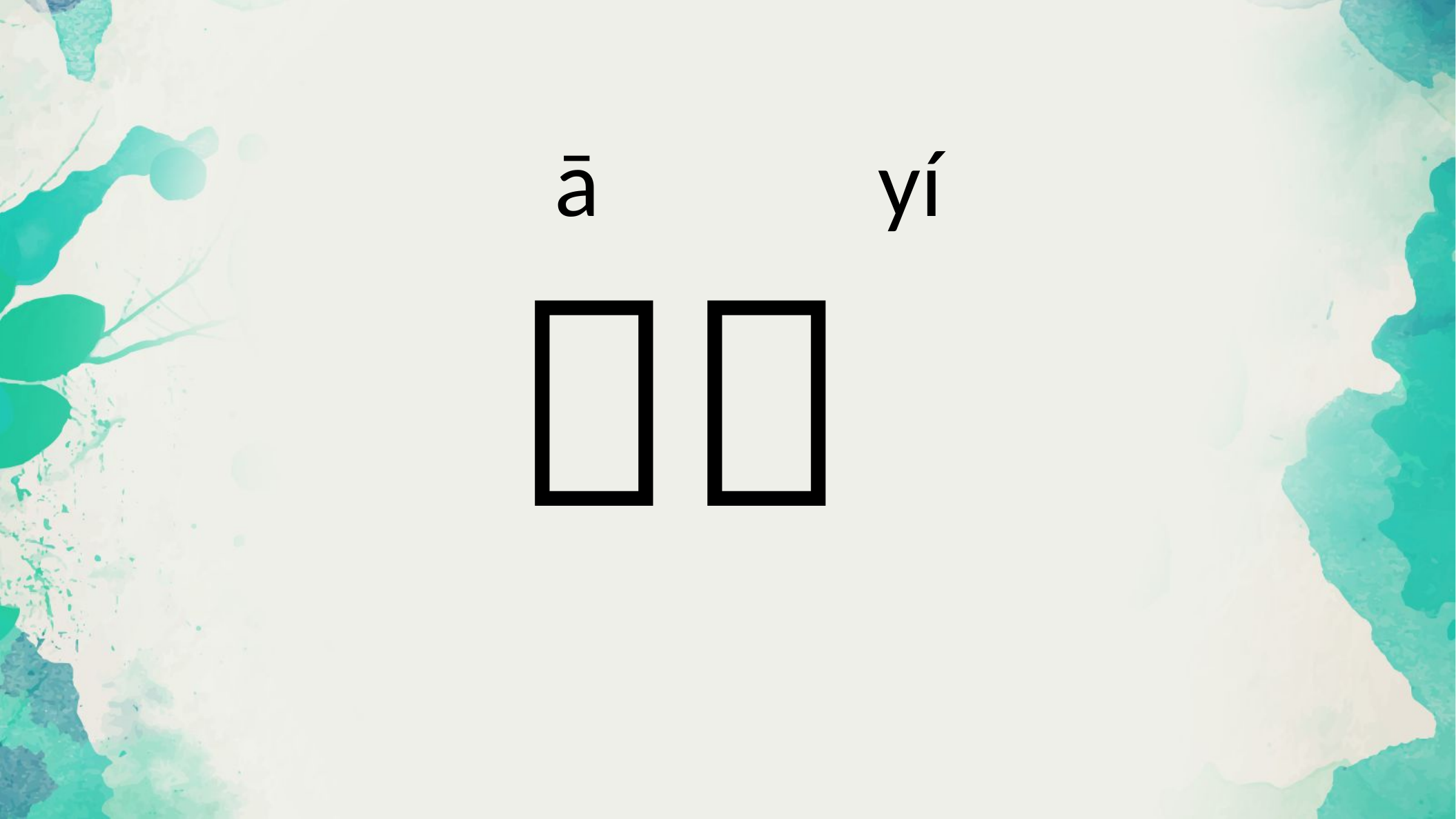

ā yí
阿姨

## Slide 8
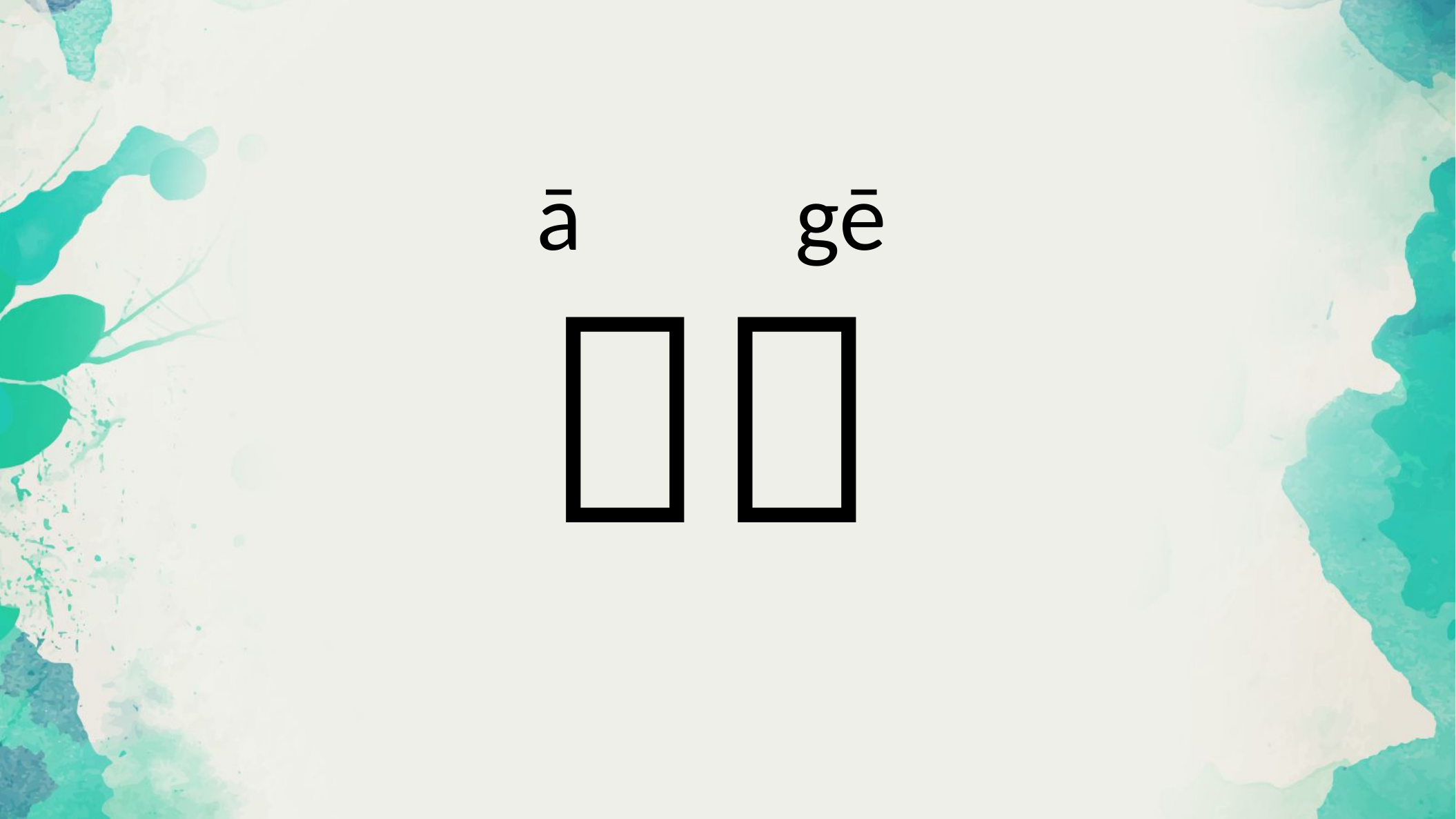

ā gē
阿哥

## Slide 9
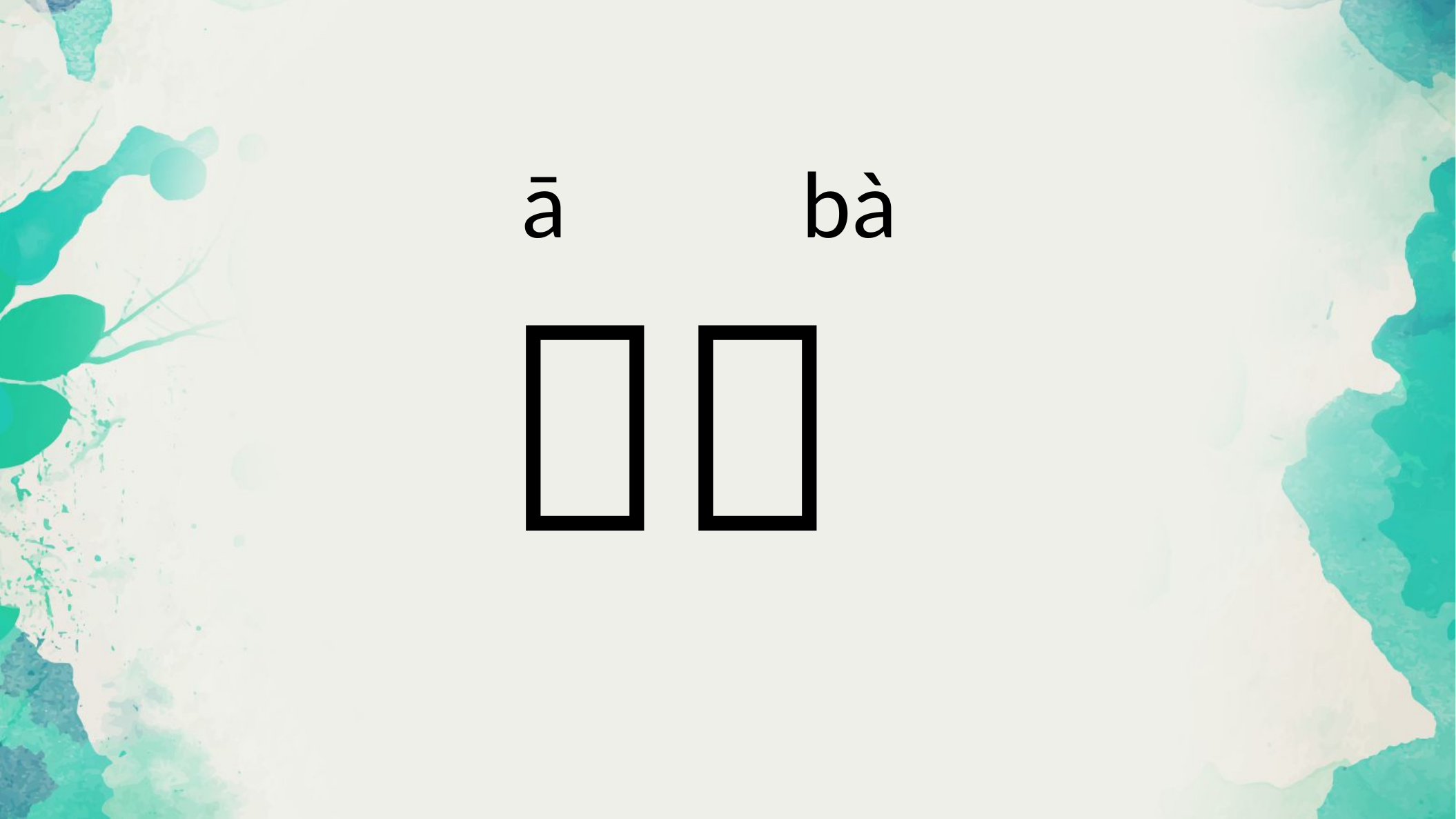

ā bà
阿爸

## Slide 10
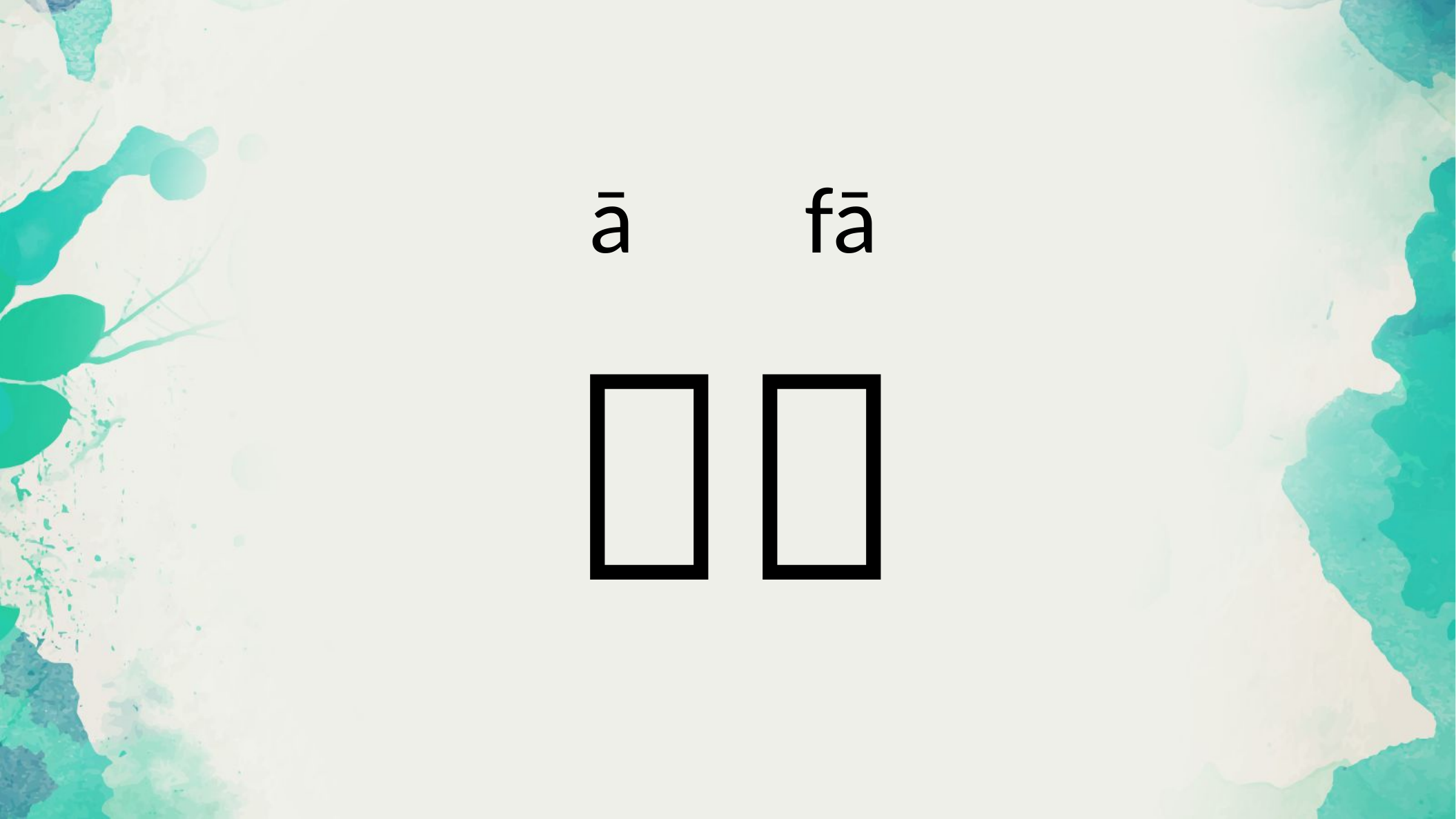

ā fā
阿发

## Slide 11
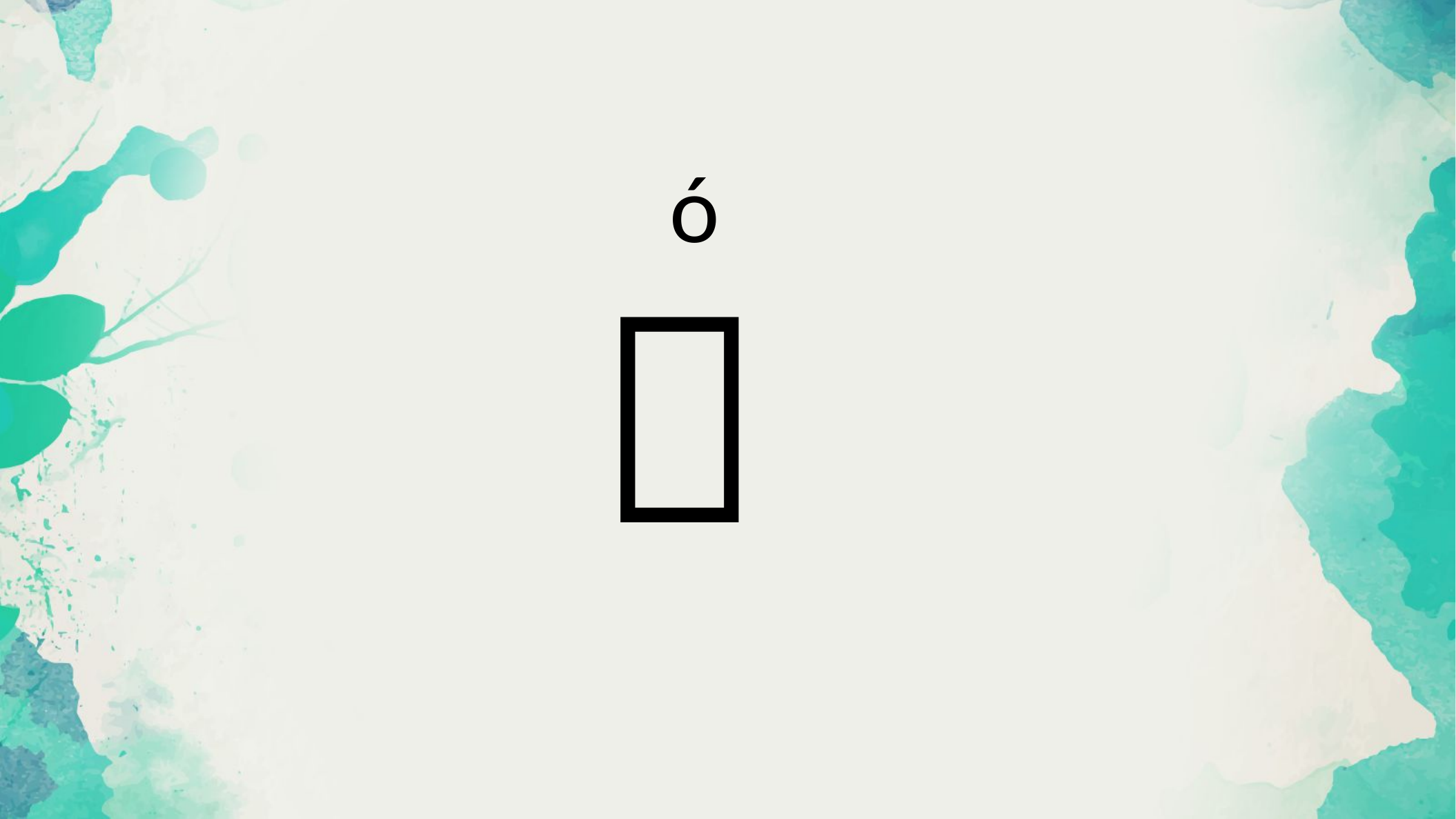

ó
哦

## Slide 12
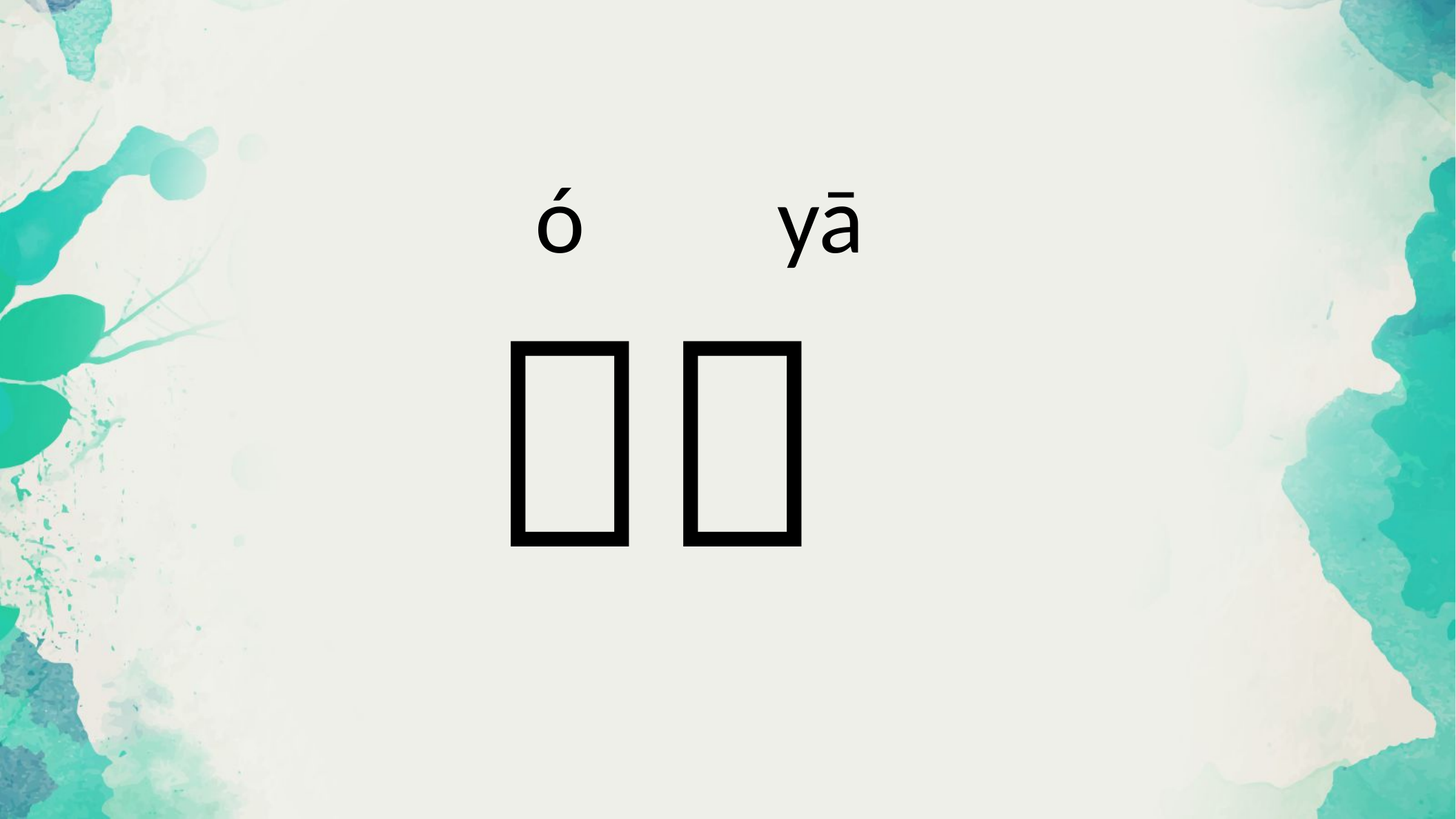

ó yā
哦呀

## Slide 13
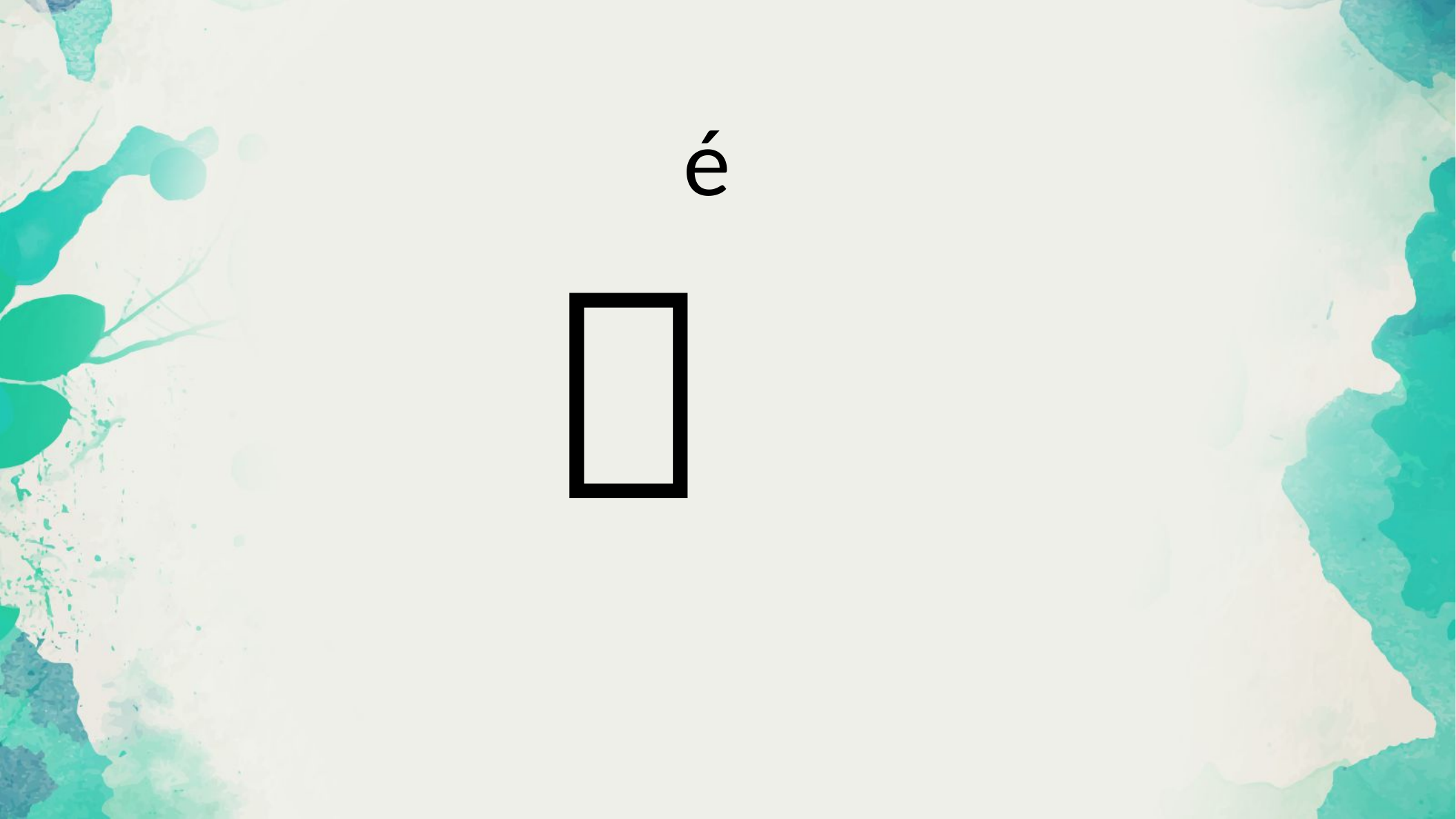

é
峨

## Slide 14
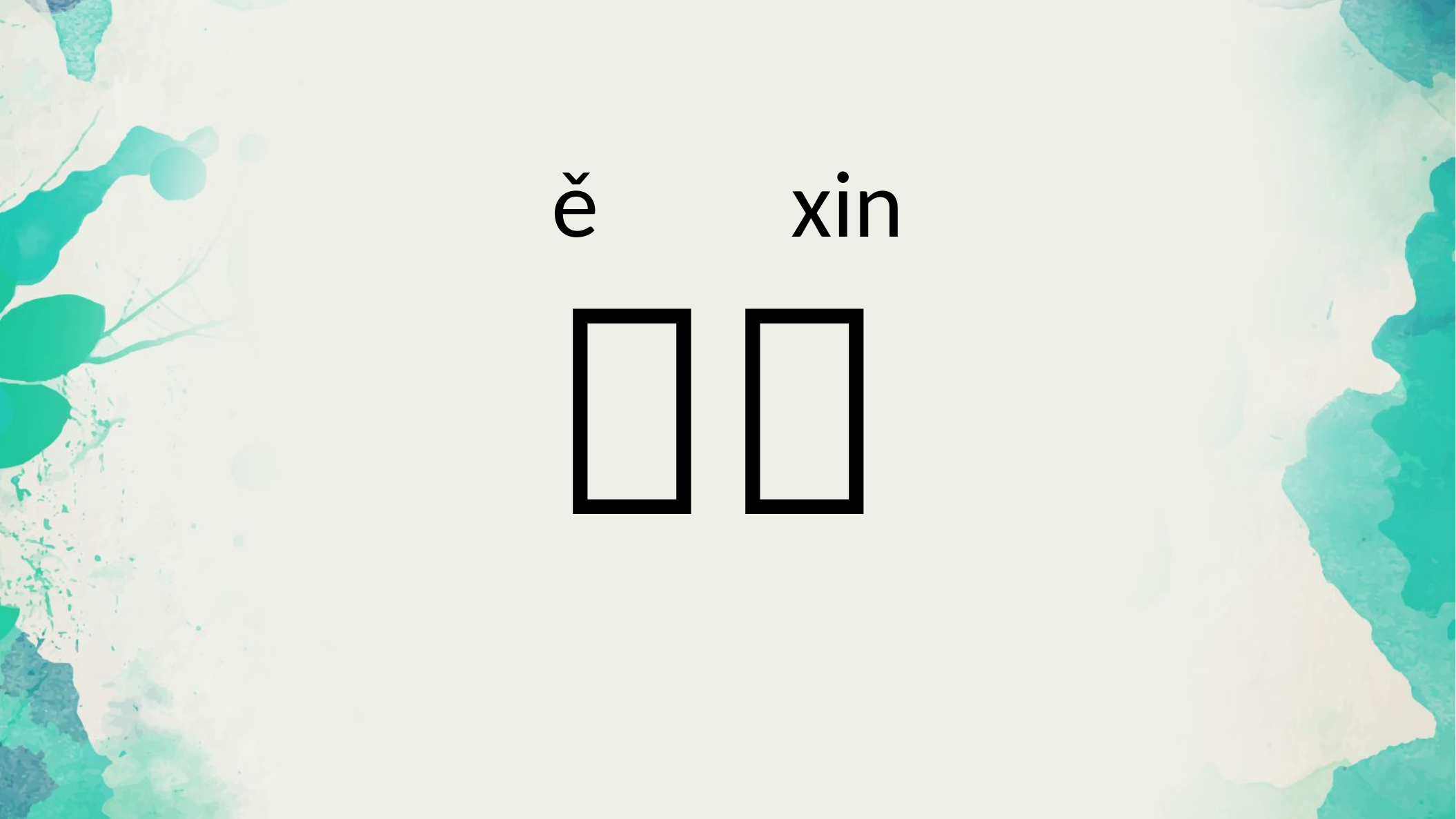

ě xin
恶心

## Slide 15
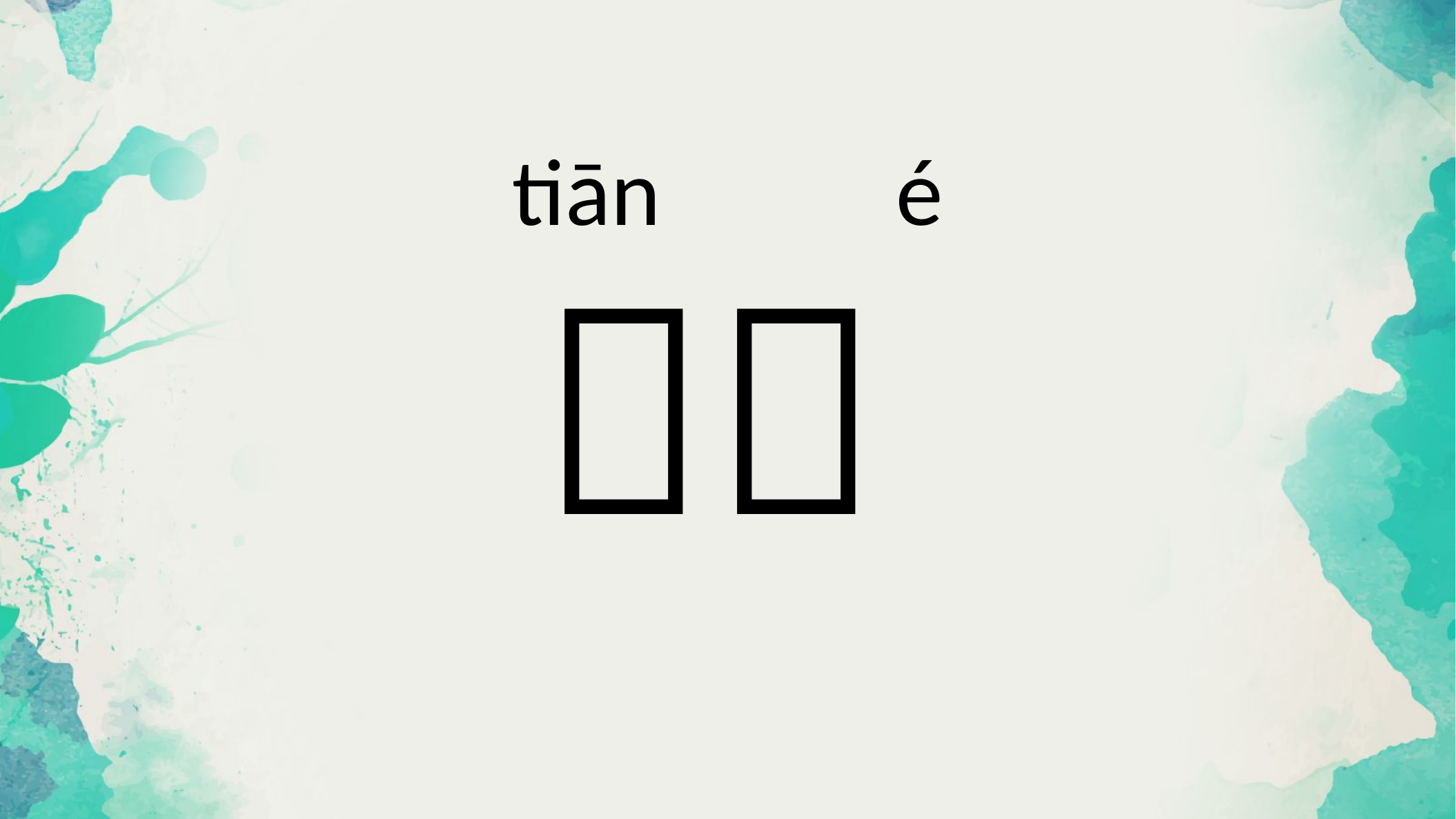

tiān é
天鹅

## Slide 16
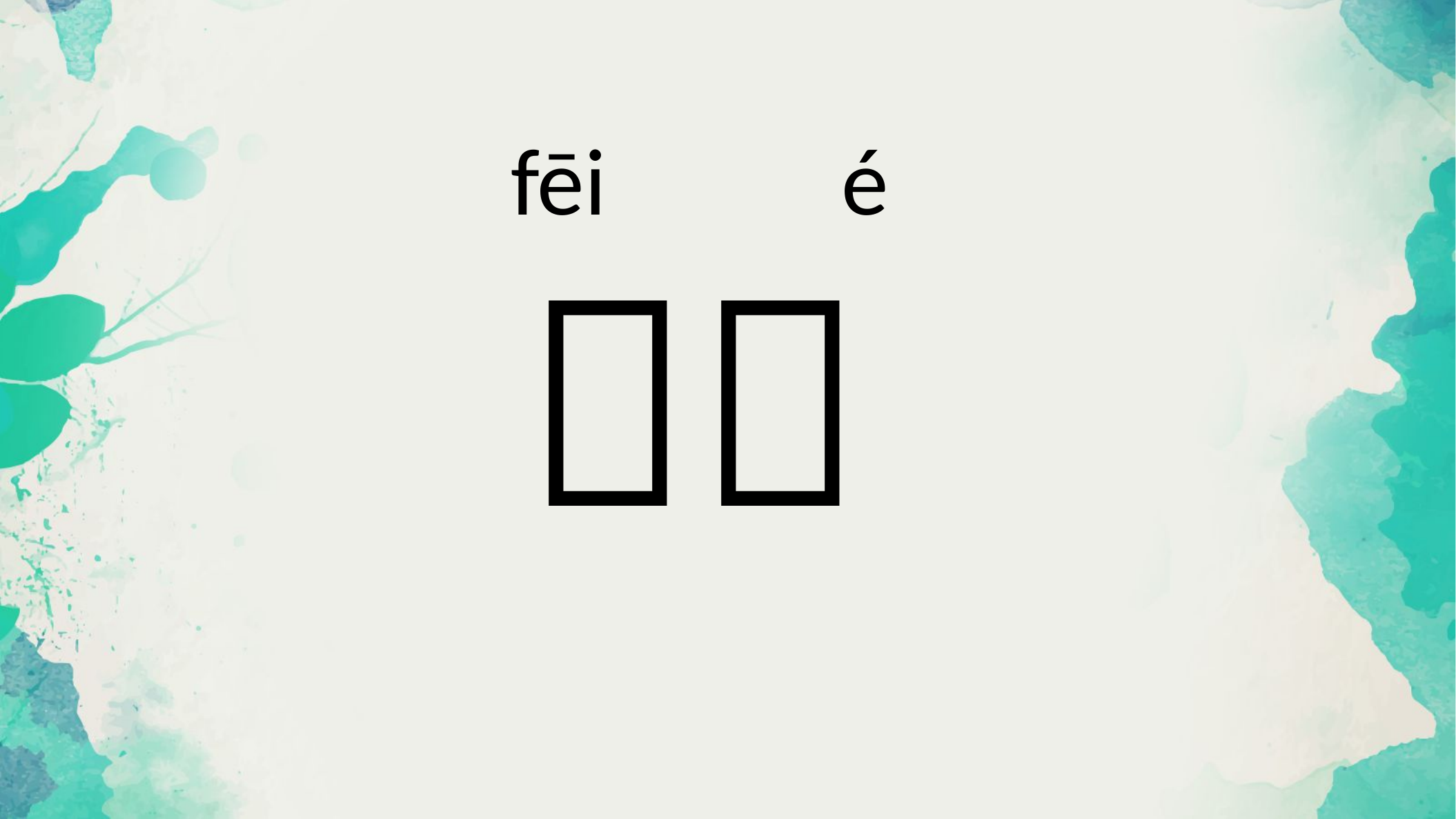

fēi é
飞蛾

## Slide 17
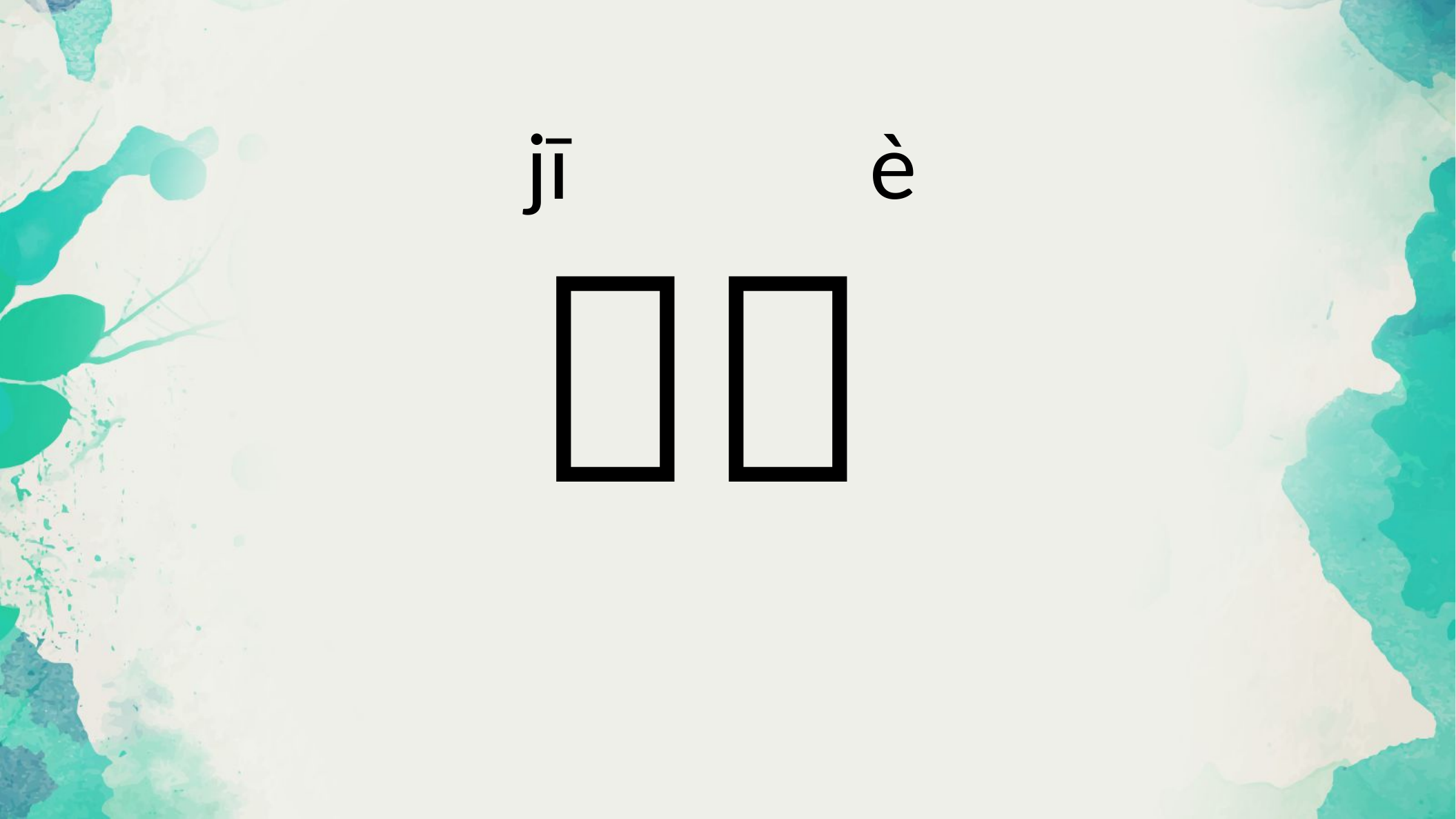

jī è
饥饿

## Slide 18
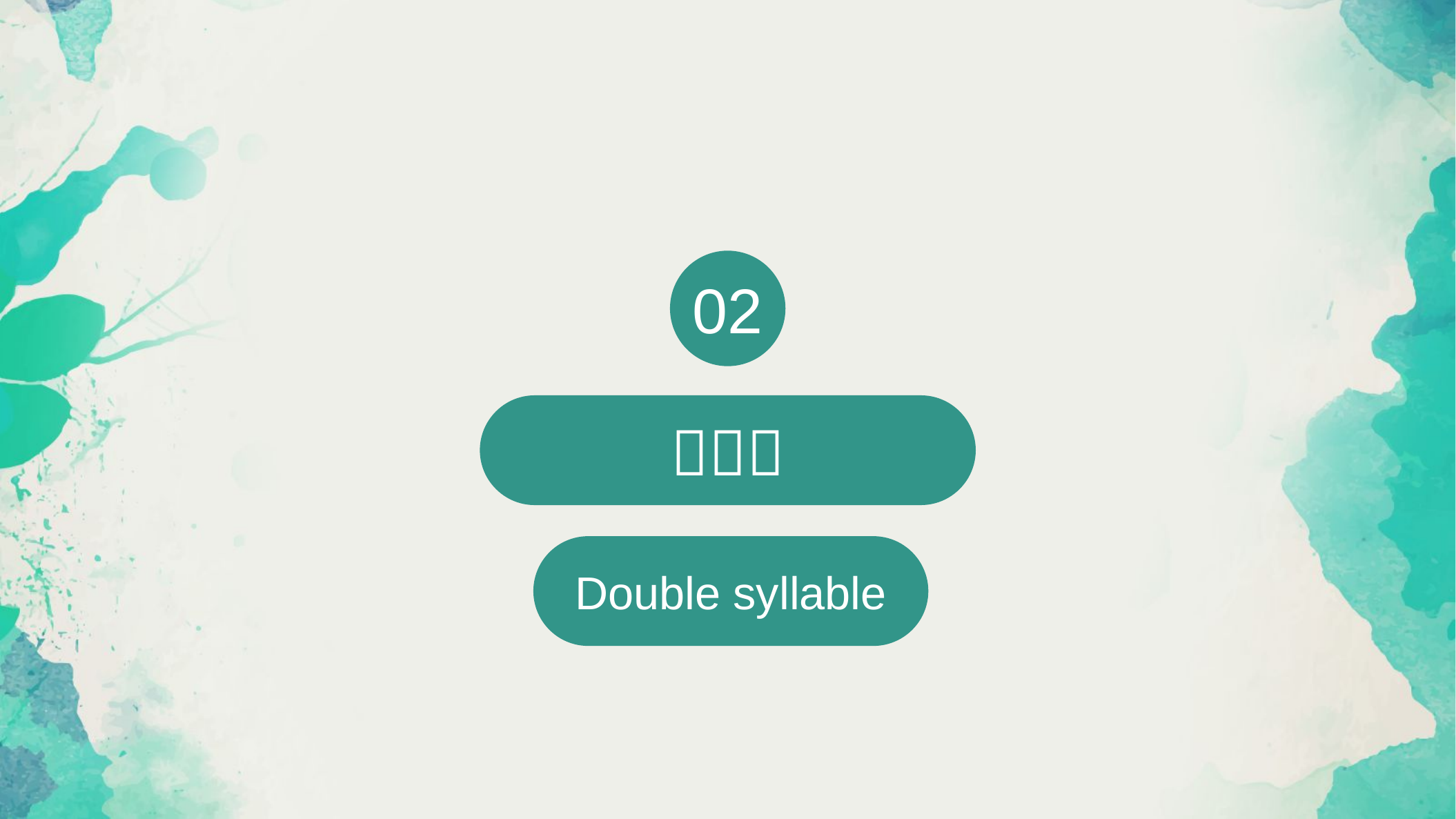

02
双音节
Double syllable

## Slide 19
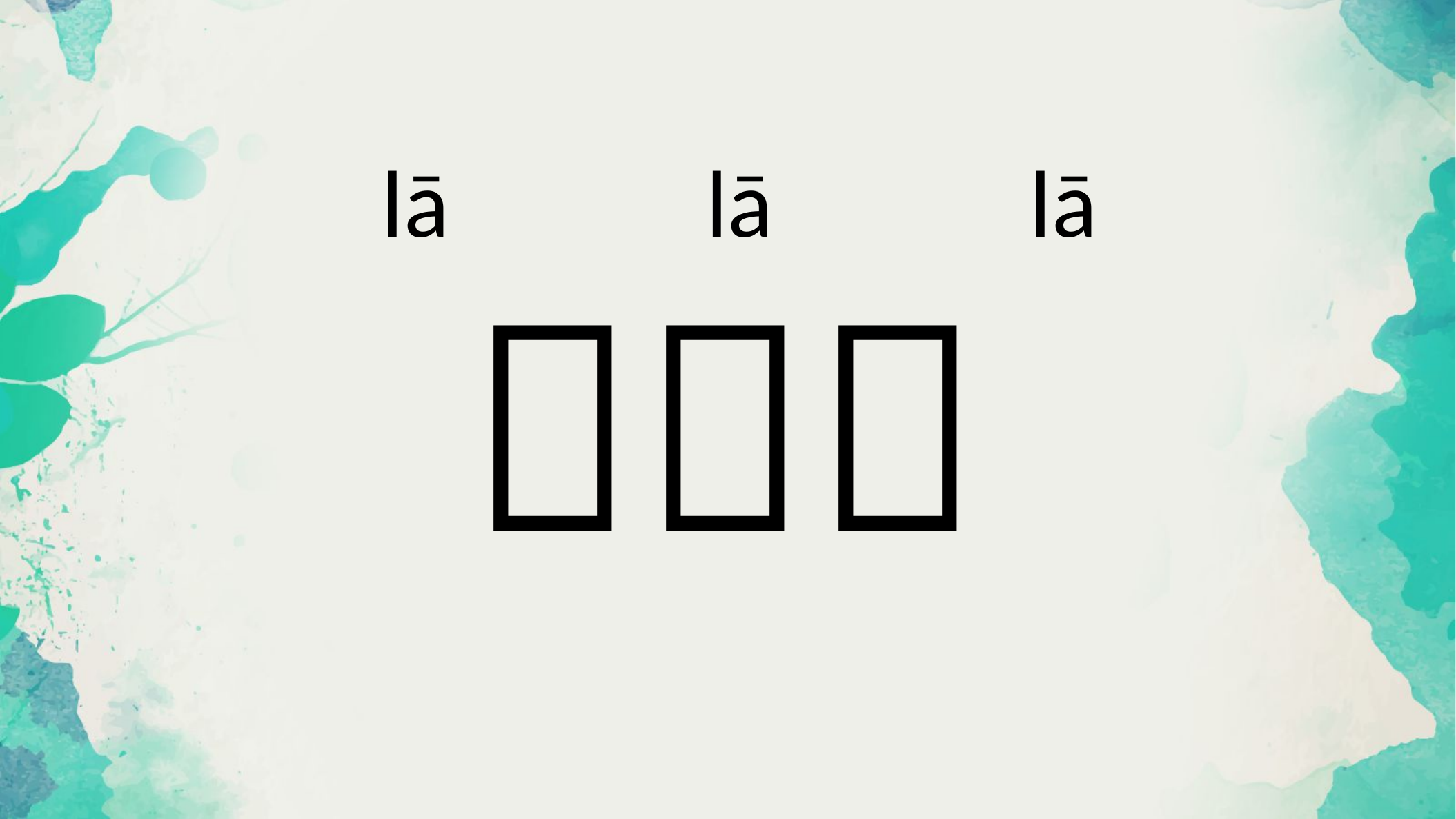

lā lā lā
啦啦啦

## Slide 20
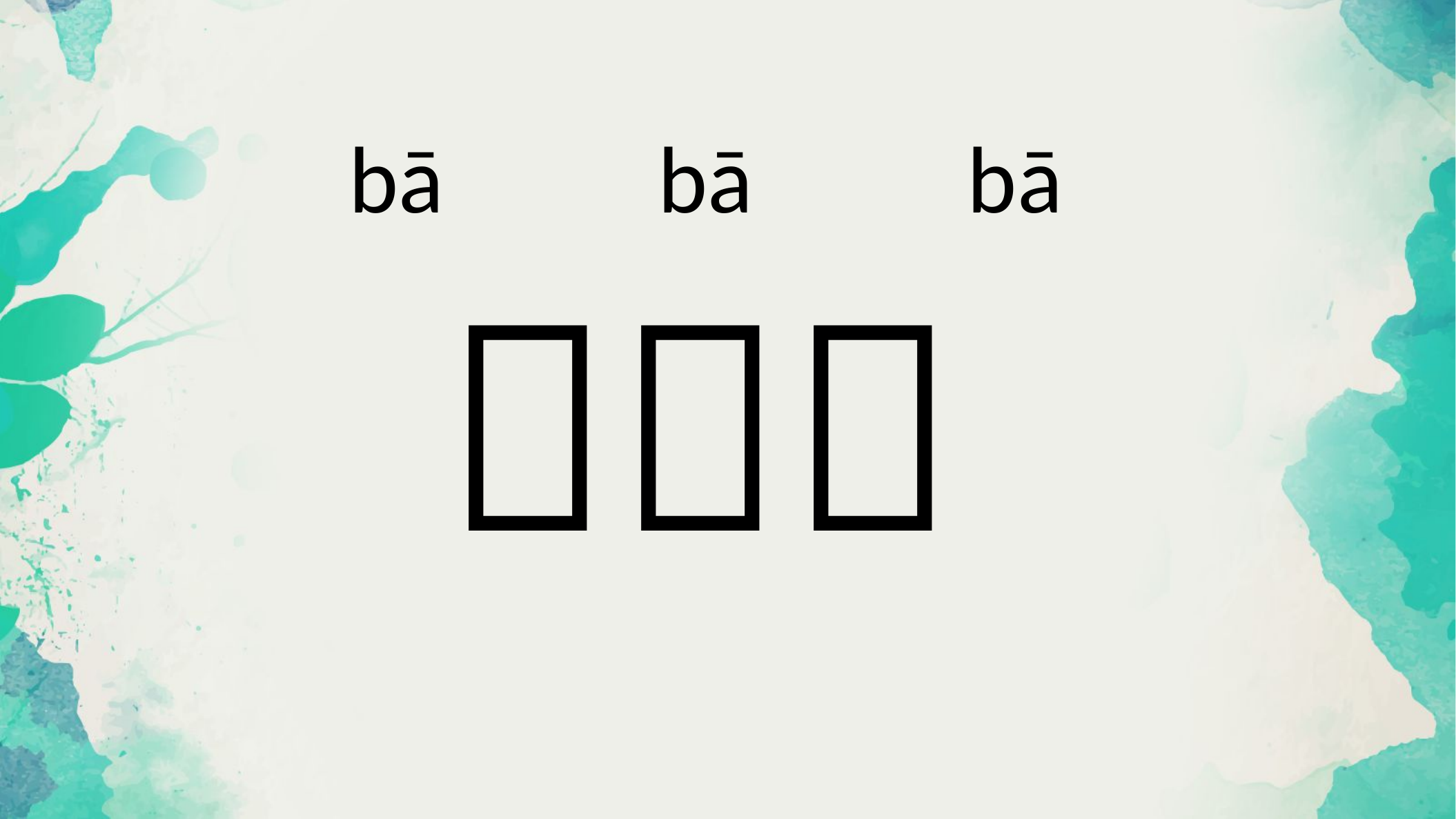

bā bā bā
叭叭叭

## Slide 21
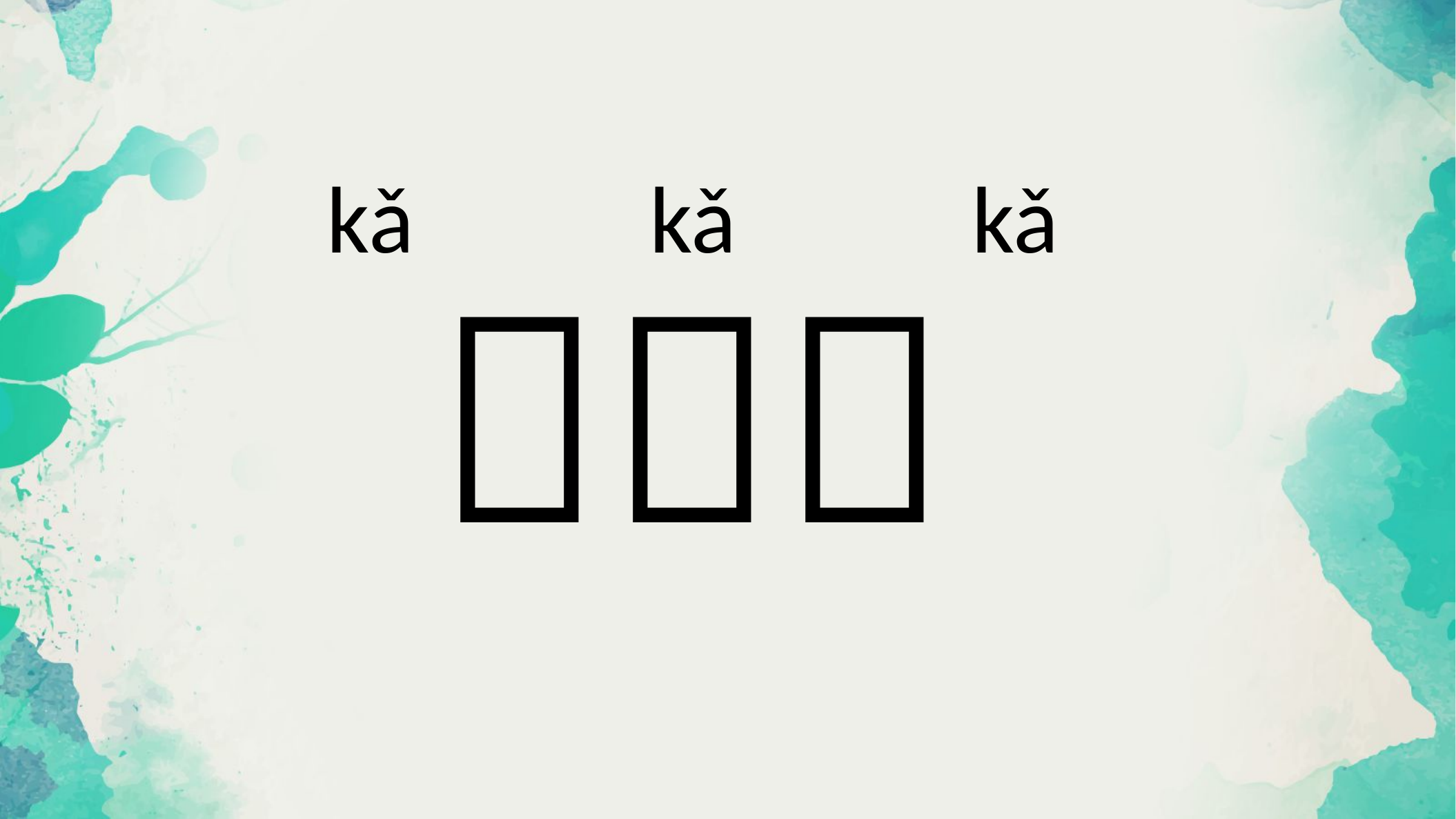

kǎ kǎ kǎ
咔咔咔

## Slide 22
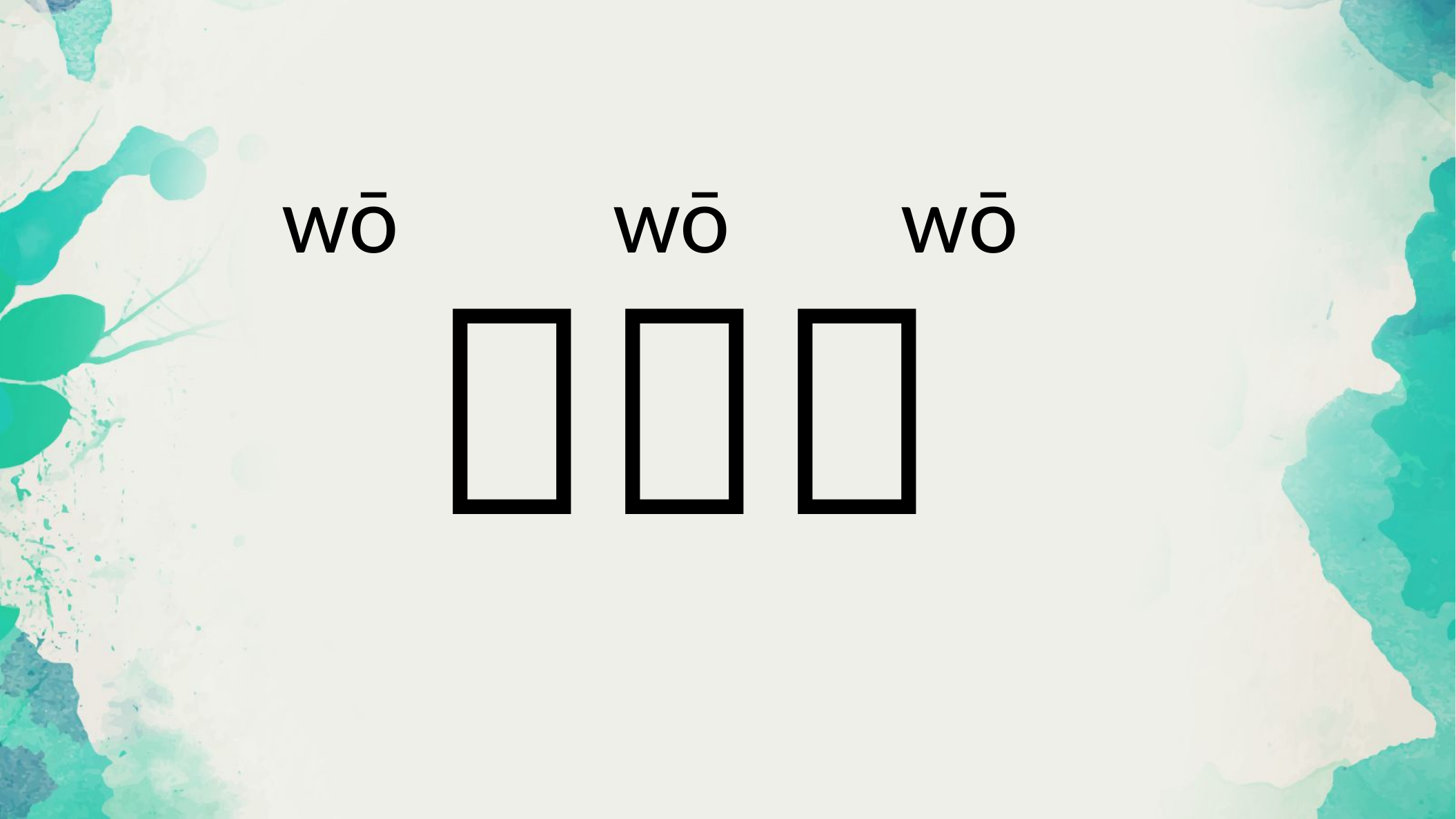

wō wō wō
喔喔喔

## Slide 23
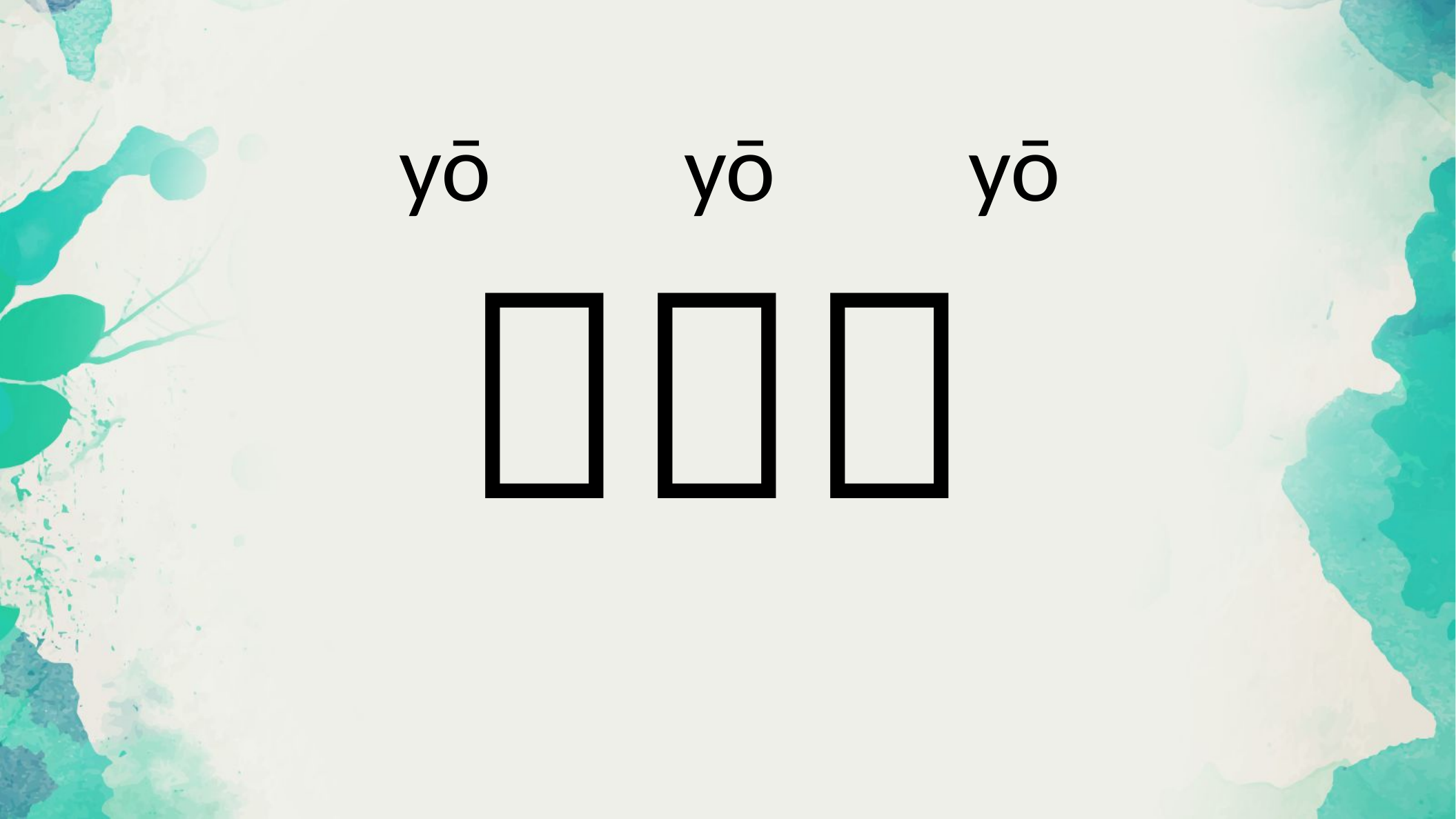

yō yō yō
哟哟哟

## Slide 24
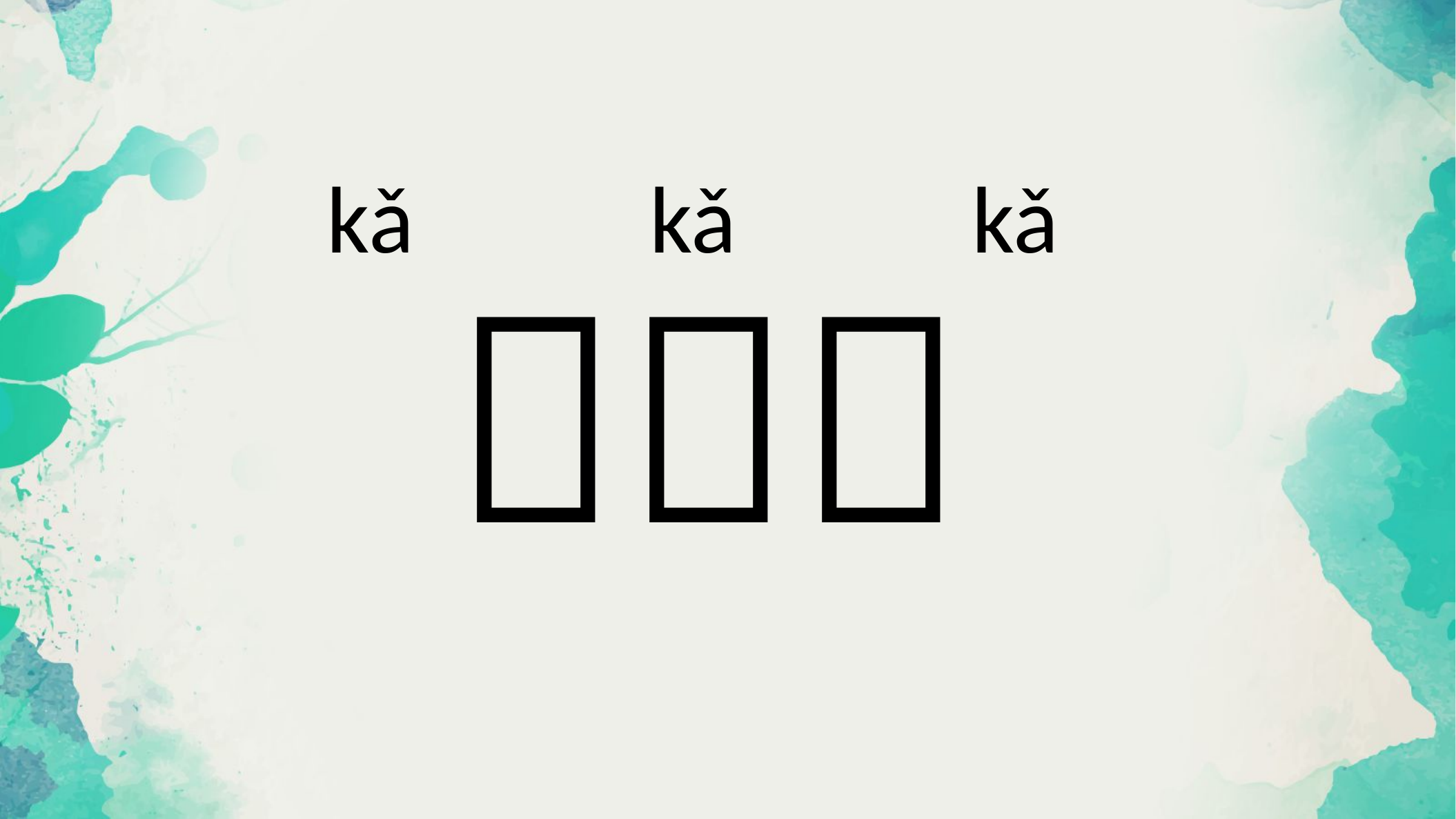

kǎ kǎ kǎ
了了了

## Slide 25
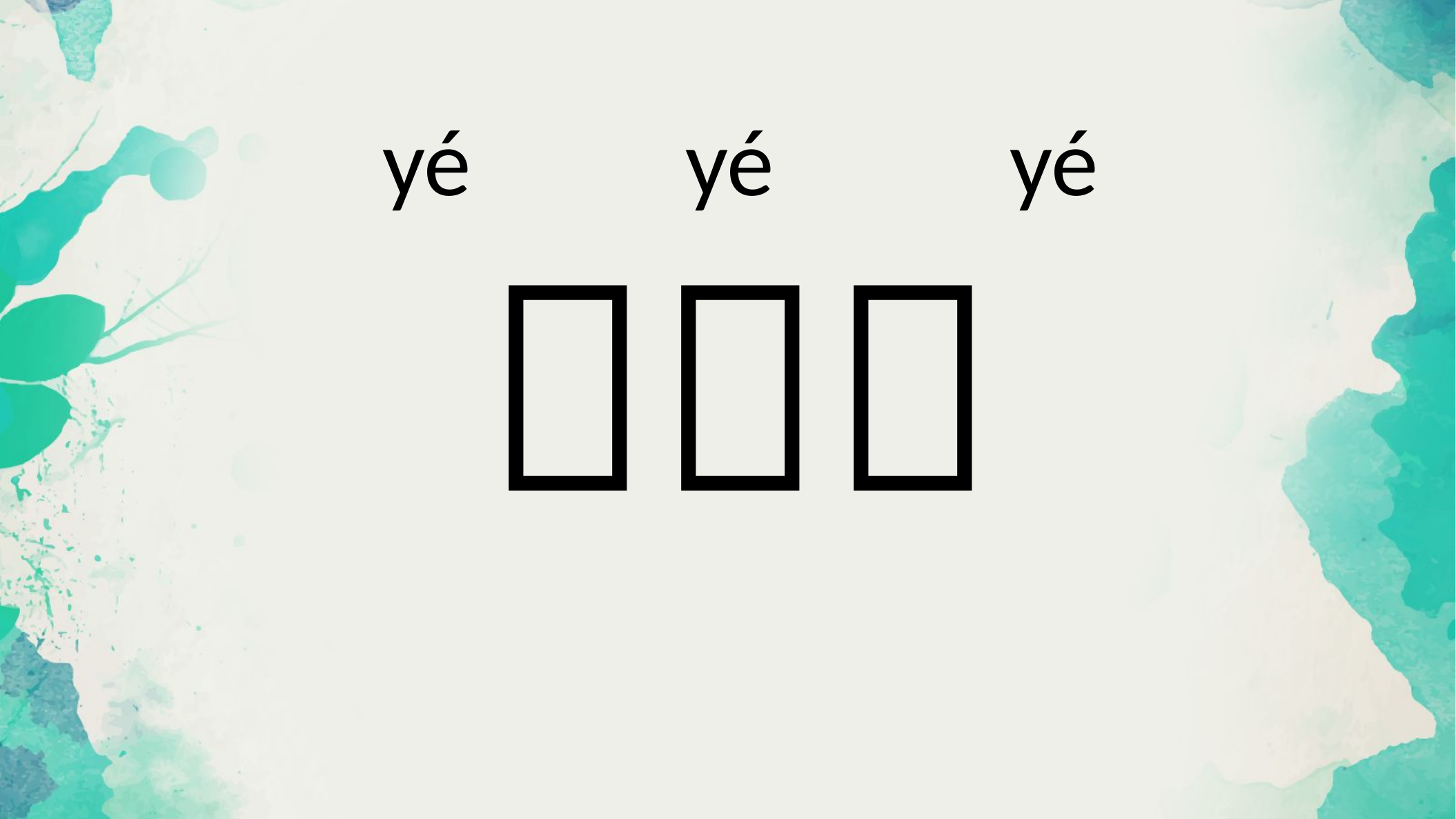

yé yé yé
耶耶耶

## Slide 26
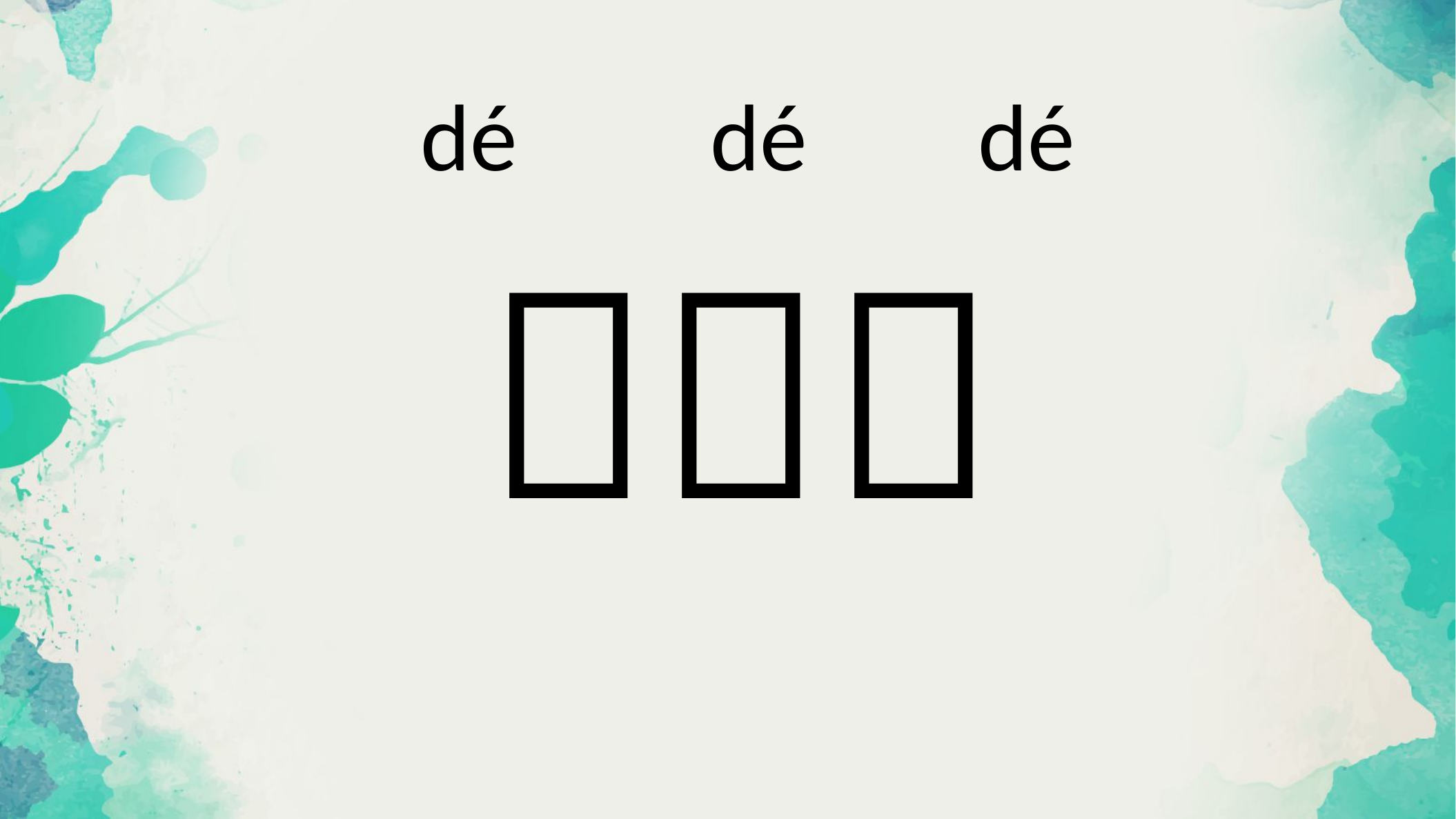

dé dé dé
得得得

## Slide 27
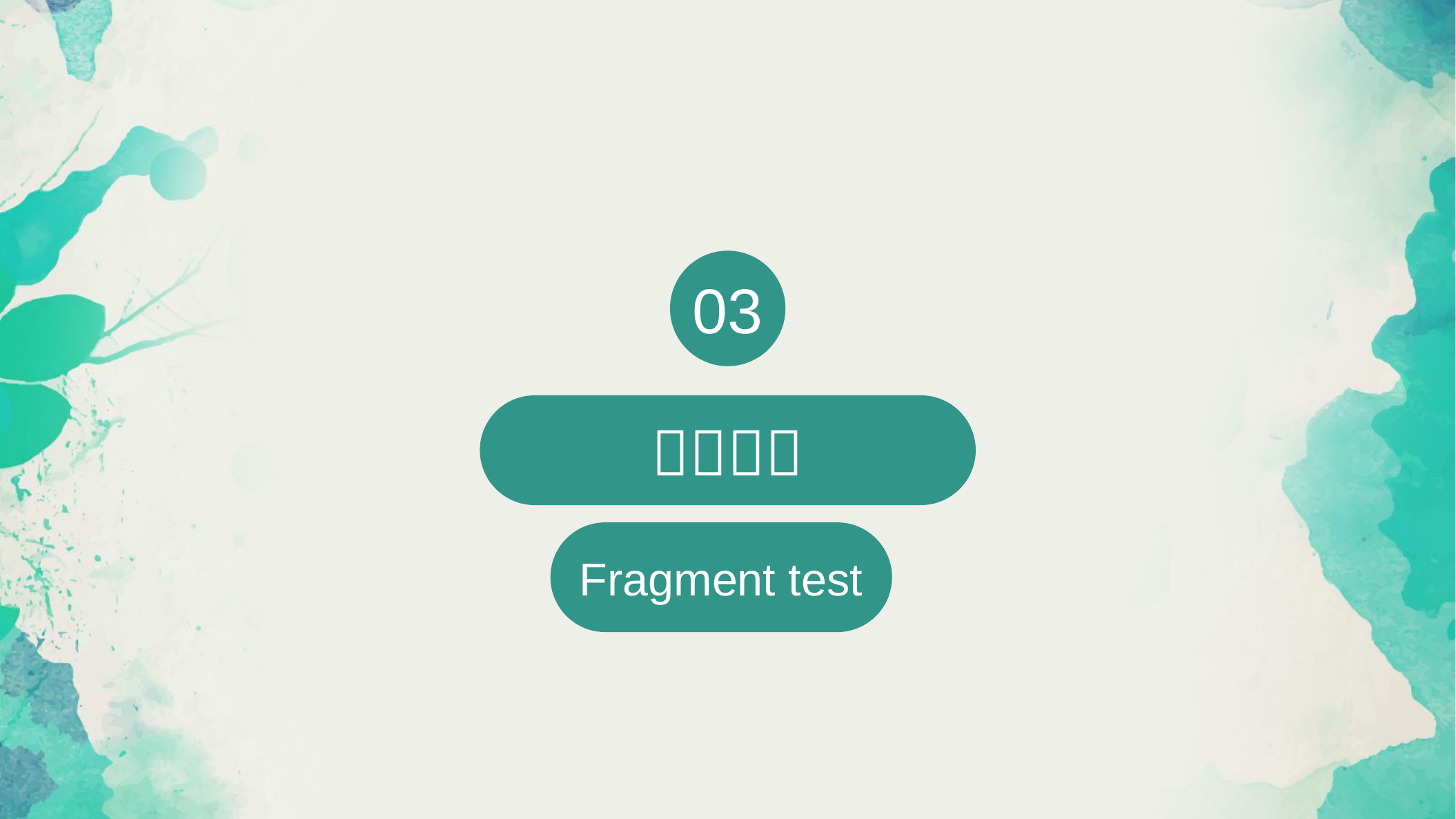

03
片段测试
Fragment test

## Slide 28
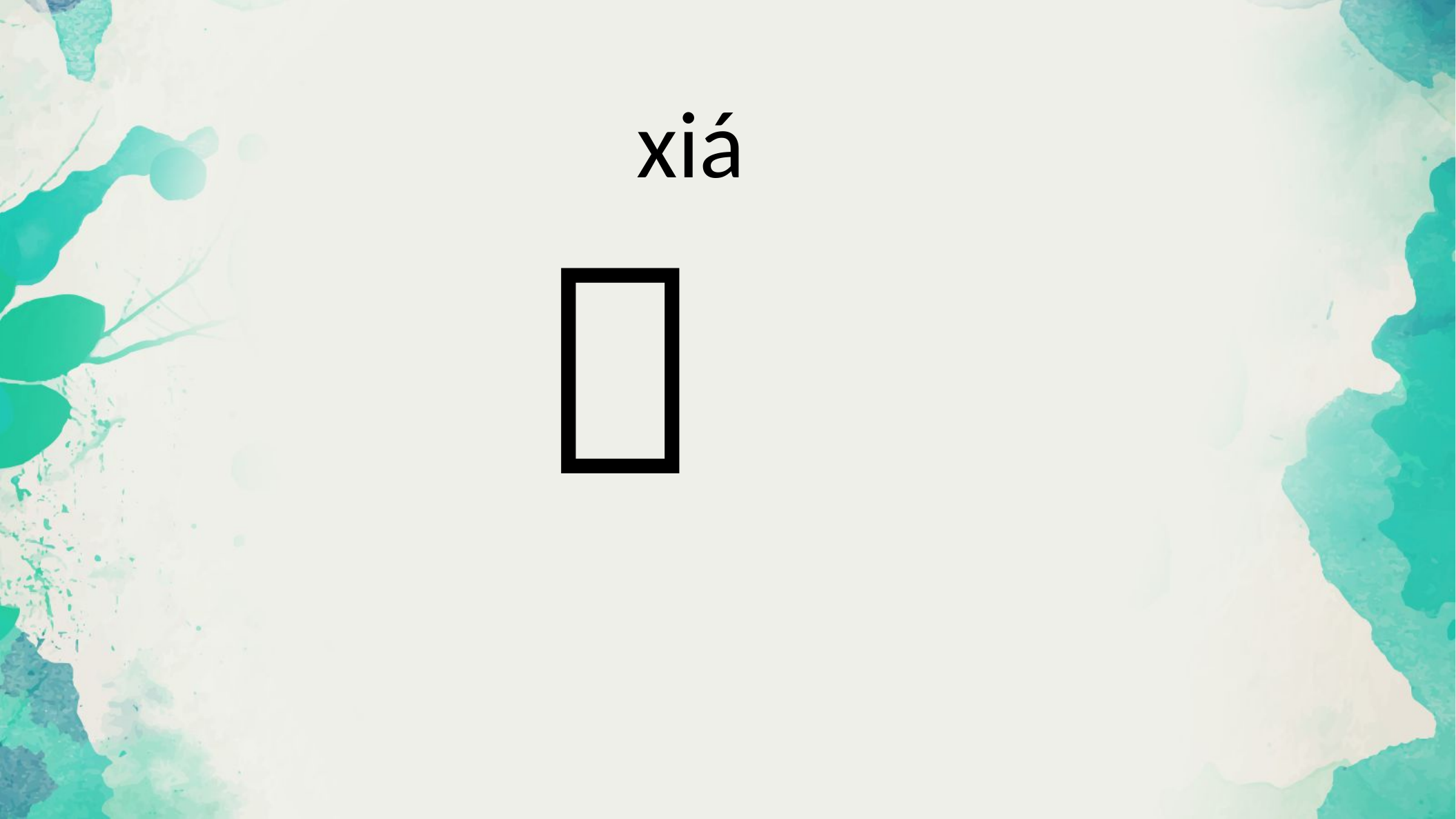

xiá
霞

## Slide 29
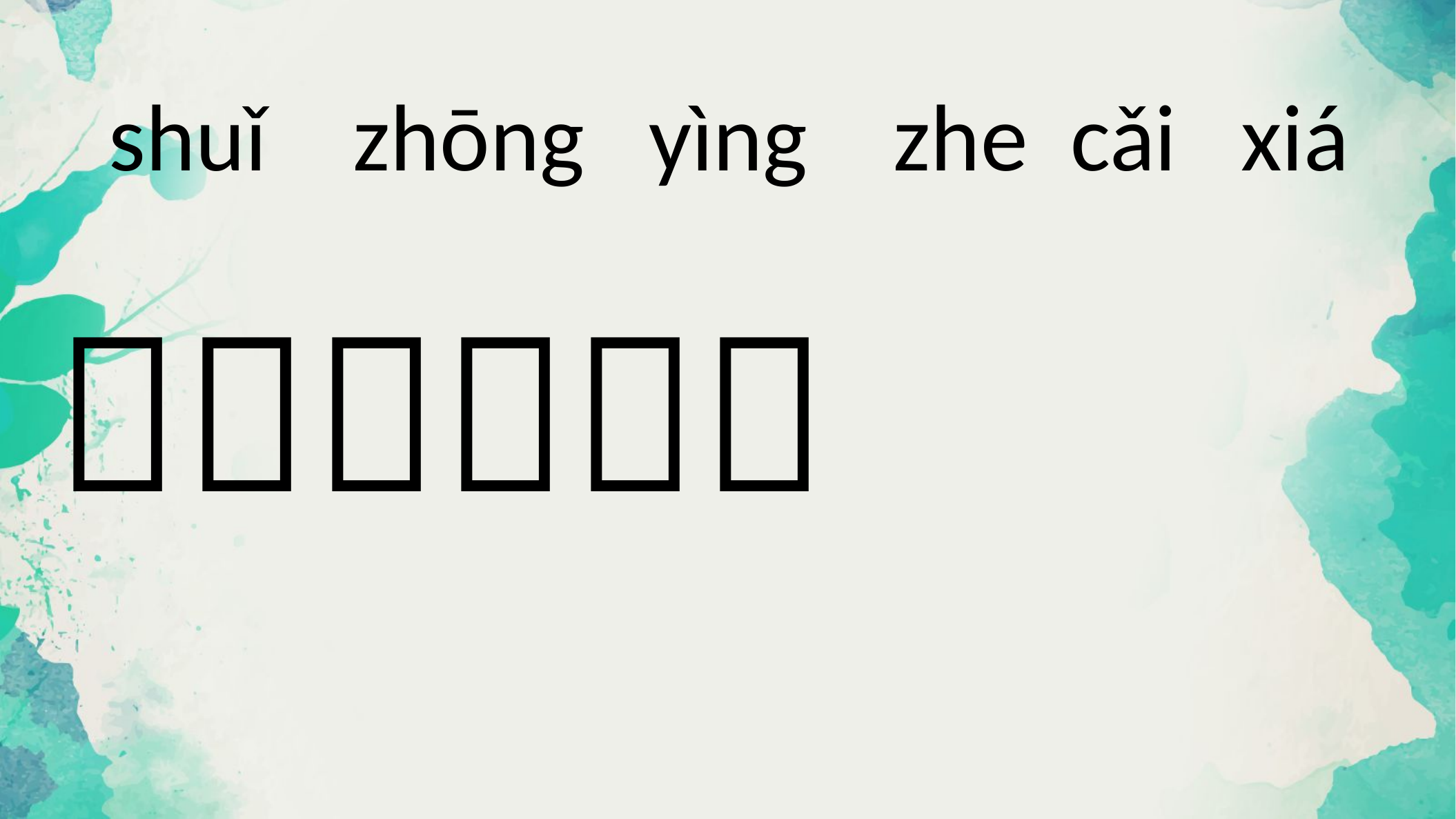

shuǐ zhōng yìng zhe cǎi xiá
水中映着彩霞

## Slide 30
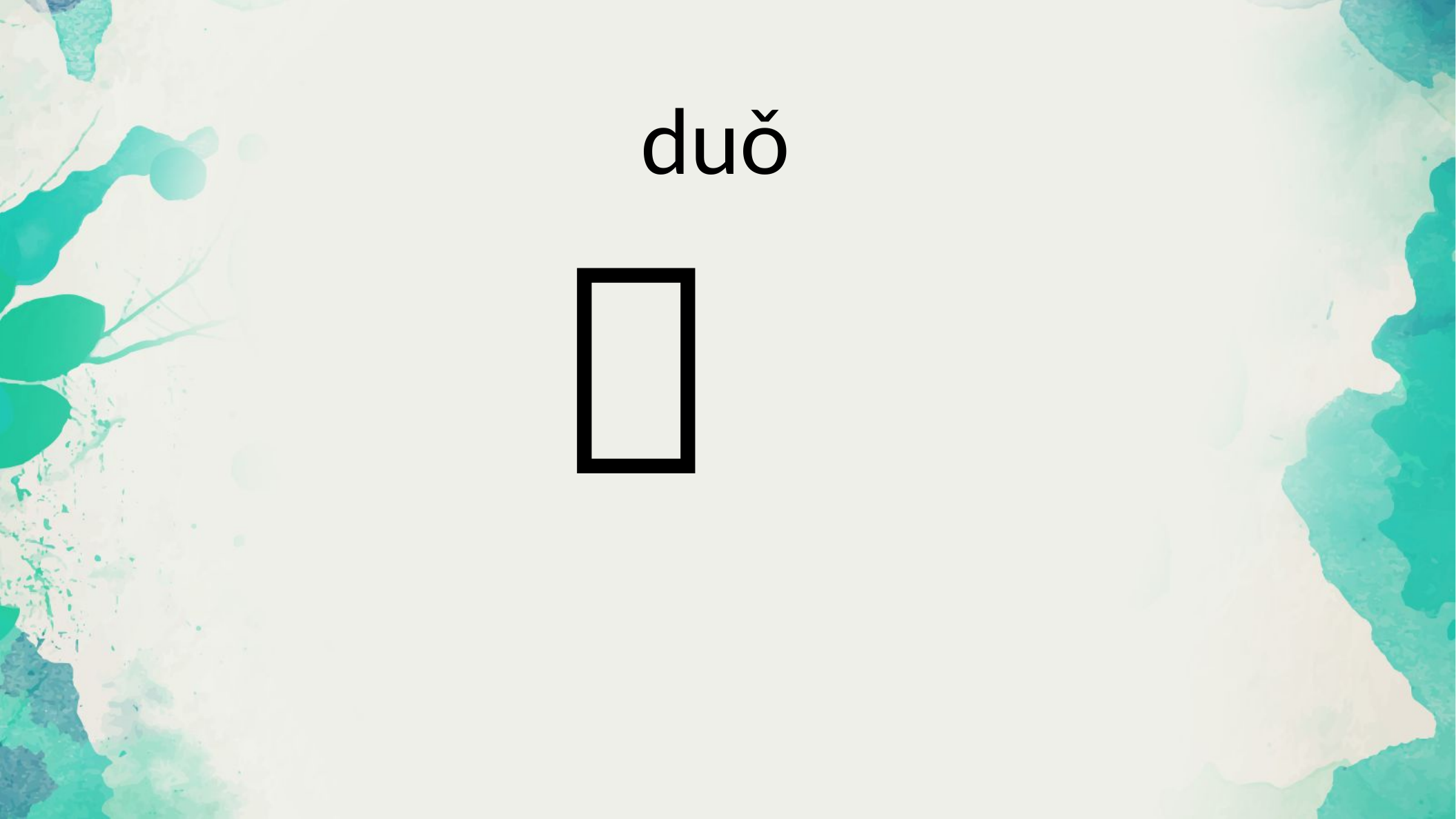

duǒ
朵

## Slide 31
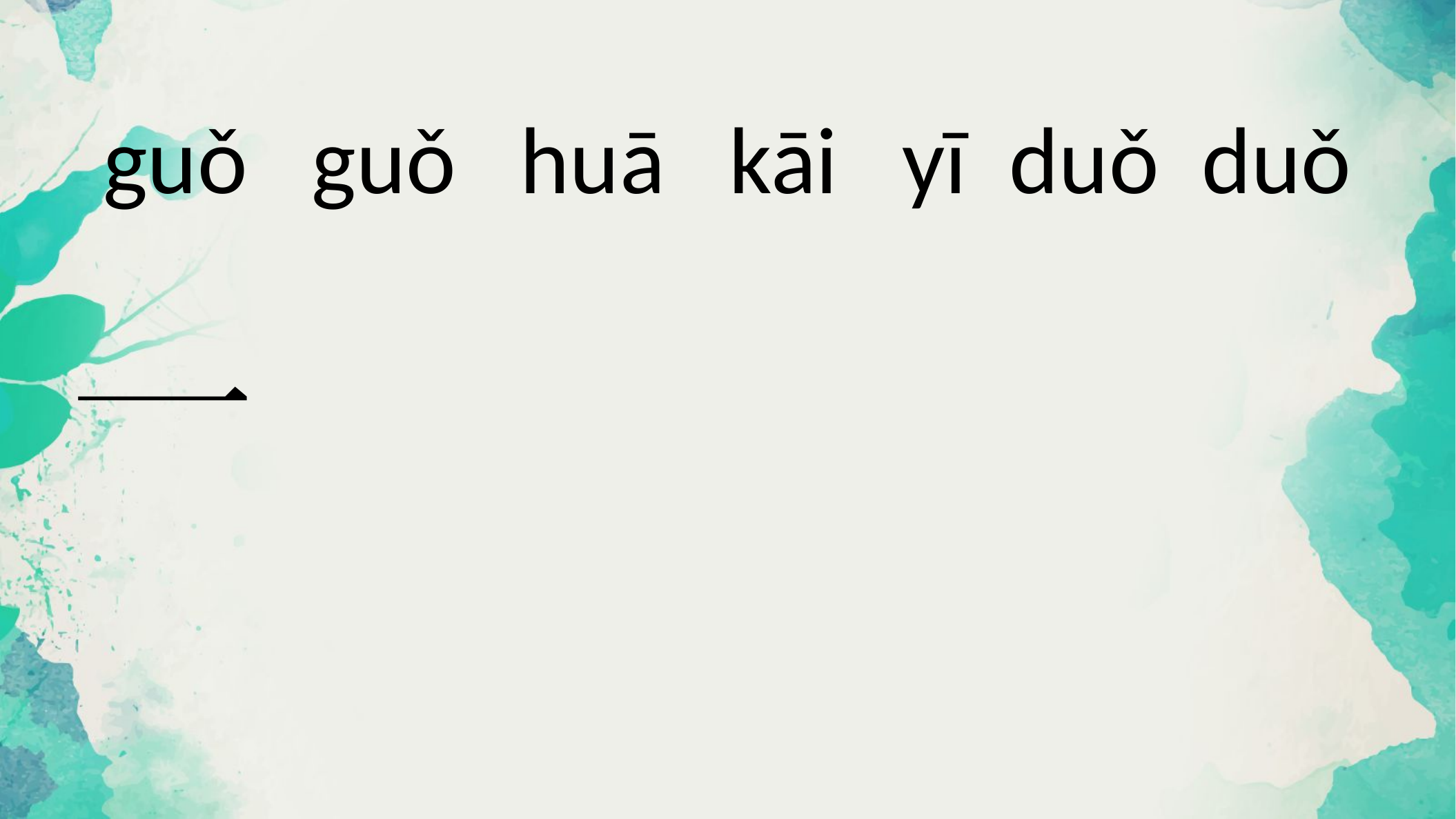

guǒ guǒ huā kāi yī duǒ duǒ
果果开花一朵朵

## Slide 32
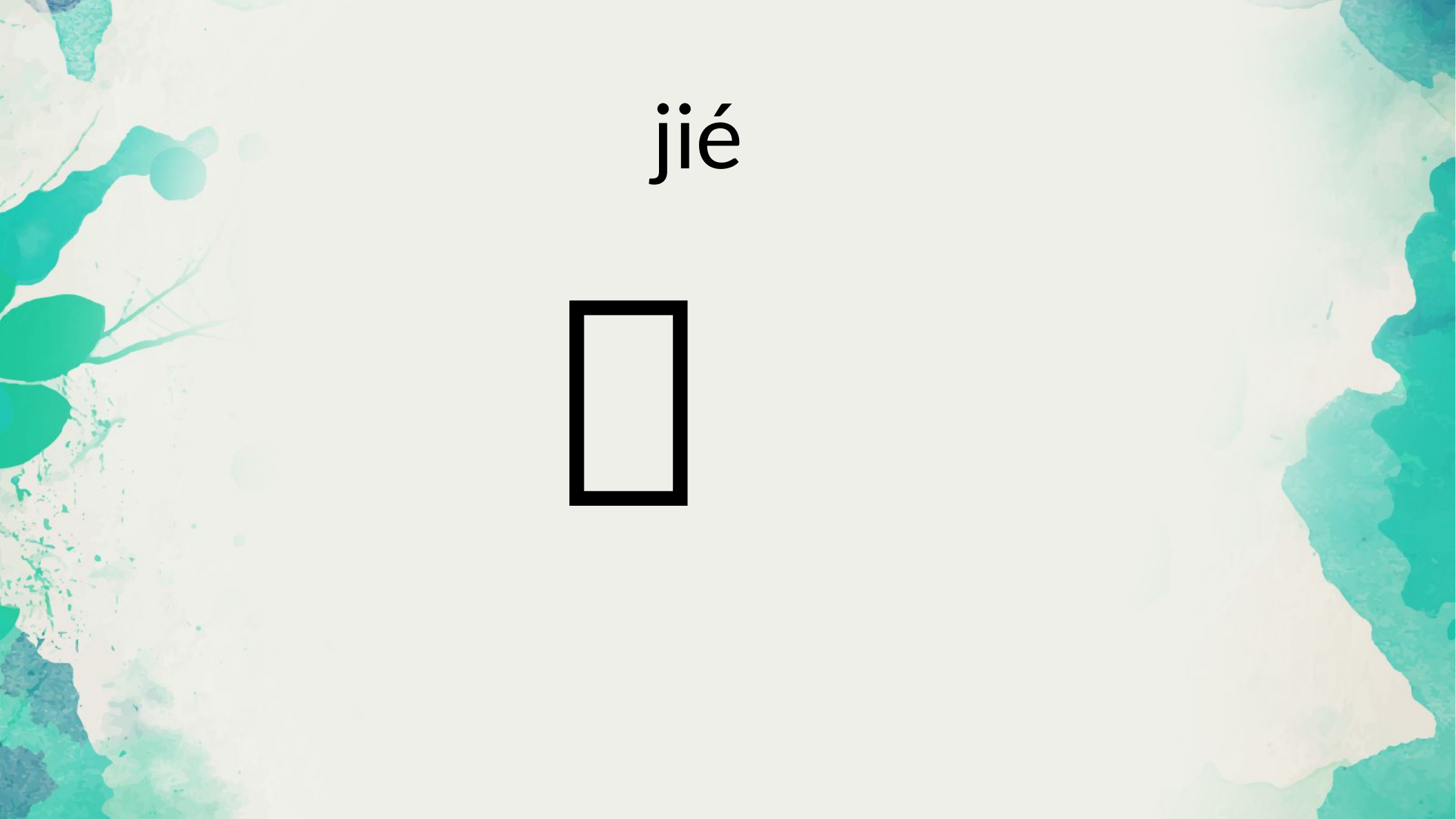

jié
洁

## Slide 33
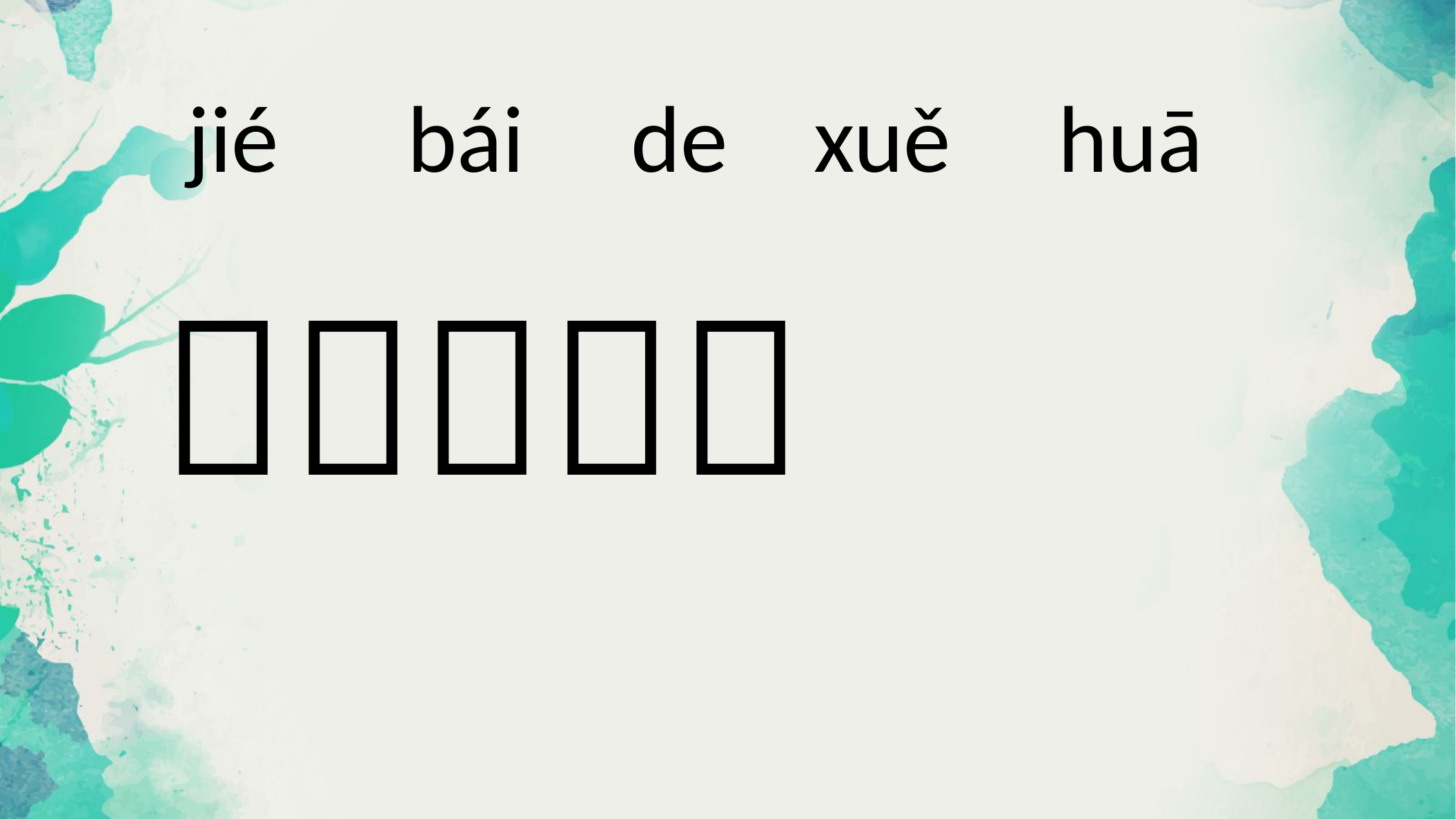

jié bái de xuě huā
洁白的雪花

## Slide 34
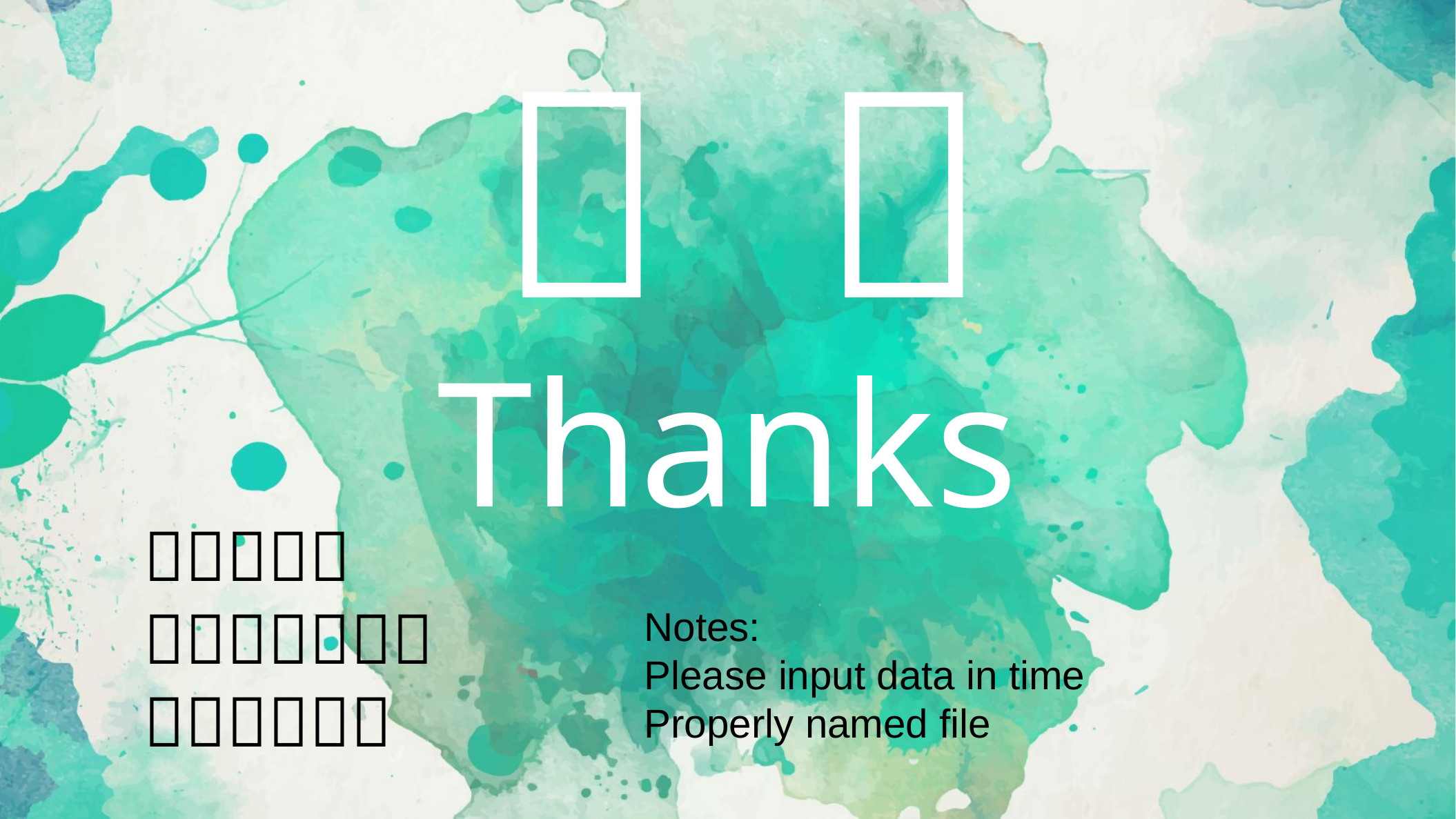

谢 谢
Thanks
注意事项：
请及时录入数据
正确命名文件
Notes:
Please input data in time
Properly named file
